# Gegen-Sangshen oral liquid and its active fractions mitigate alcoholic liver disease in mice through repairing intestinal epithelial injury and regulating gut microbiota

**DOI:** 10.1186/s13020-024-01049-y

**Published:** 2024-12-23

**Authors:** Shulin Wei, Mingxing Li, Long Zhao, Tiangang Wang, Ke Wu, Jiayue Yang, Yubin Liu, Yueshui Zhao, Fukuan Du, Yu Chen, Shuai Deng, Jing Shen, Zhangang Xiao, Wanping Li, Xiaobing Li, Yuhong Sun, Li Gu, Mei Wei, Zhi Li, Xu Wu

**Affiliations:** 1 Cell Therapy & Cell Drugs of Luzhou Key Laboratory, Department of Pharmacology, School of Pharmacy, Southwest Medical University, Luzhou, 646000 Sichuan China; 2 South Sichuan Institute of Translation Medicine, Luzhou, 646000 Sichuan China; 3 Department of Spleen and Stomach Diseases, The Affiliated Traditional Chinese Medicine Hospital, Southwest Medical University, Luzhou, 646000 Sichuan China; 4 School of Pharmacy, Sichuan College of Traditional Chinese Medicine, Mianyang, 621000 Sichuan China; 5 Gulin County Hospital of Traditional Chinese Medicine, Luzhou, 646500 Sichuan China; 6 The Key Laboratory of Integrated Traditional Chinese and Western Medicine for Prevention and Treatment of Digestive System Diseases of Luzhou City, The Affiliated Traditional Chinese Medicine Hospital, Southwest Medical University, Luzhou, 646000 Sichuan China; 7 Department of Paediatrics, & Department of Paediatric Care, Luzhou People’s Hospital, Luzhou, 646000 Sichuan China

**Keywords:** Alcoholic liver disease, Gegen-Sangshen oral liquid, Gut-liver axis, Intestinal organoid, Gut microbiota

## Abstract

**Background:**

Liuweizhiji Gegen-Sangshen oral liquid (LGS), as a Chinese medicinal preparation, is developed from a Traditional Chinese medicinal formula consisting of six Chinese medicinal herbs, including *Puerariae lobatae* radix, *Hoveniae* semen, *Imperatae* rhizoma, *Crataegi* fructus, *Mori* fructus and *Canarli* fructus, and has been extensively utilized in the prevention and treatment of alcoholic liver disease (ALD) clinically. Previous study has demonstrated that LGS dose-dependently mitigated ALD in rat models. However, whether and how the main characteristic constituents of LGS (the flavonoid and polysaccharide fractions, LGSF and LGSP) contribute to the anti-ALD effect remains unclear. This study aimed to assess the anti-ALD effect of LGS and its main fractions (LGSF and LGSP) in a murine model of ALD and to explore the underlying mechanisms.

**Methods:**

ALD mouse model was constructed using the chronic and binge ethanol feeding method. Biochemical determinations of AST, ALT, TC, TG, ADH, ALDH, HDL, LDL, IL-1β, IL-6, and TNF-α were performed using corresponding kits. Histopathological examination of liver and intestinal sections was conducted based on the H&E staining. Lipid accumulation in hepatocytes was evaluated by oil red O staining. Ethanol metabolism was assessed by determining the activity of ADH and ALDH enzymes. Intestinal barrier function was analyzed based on immunohistochemistry analysis of ZO-1 and occludin and immunofluorescence analysis of epithelial markers, Lgr5, Muc2, and Lyz1. Intestinal epithelial apoptosis was detected by TUNEL staining. Mouse fecal microbiota alterations were analyzed by 16S rRNA sequencing. An in vitro epithelial injury model was established by developing TNF-α-induced 3D-cultured intestinal organoids. In vitro culture of specific bacterial strains was performed.

**Results:**

The results showed that LGS and its flavonoid and polysaccharide fractions (LGSF and LGSP) significantly alleviated ALD in mice through attenuating hepatic injury and inflammation, improving liver steatosis and promoting ethanol metabolism. Notably, LGS, LGSP, and LGSF mitigated intestinal damage and maintained barrier function in ALD mice. The intestinal barrier protection function of LGS, LGSP, and LGSF was generally more obvious than that of the positive drug meltadosine. Further study demonstrated that LGS, LGSP, and LGSF promoted intestinal epithelial repair via promoting Lgr5^+^ stem cell mediated regeneration in TNF-α-induced intestinal organoids. LGS and LGSF, other than LGSP, had a better effect on repair of epithelial injury in vitro. Moreover, LGS, LGSP, and LGSF remarkably alleviated gut dysbiosis in ALD mice via at least partially recovery of alcohol-induced microbial changes and induction of specific bacterial groups. In vitro culture of bacterial strains indicated that LGS, LGSP, and LGSF had a specific impact on bacterial growth. LGS and LGSP, but not the LGSF, significantly promoted the growth of *Lactobacillus*. Similarly, LGS and LGSP significantly increased the proliferation of *Bacteroides sartorii*, and LGSF had a minimal effect. LGS, LGSP and LGSF all promoted the growth of *Bacillus coagulans*, *Bifidobacterium adolescentis*, and *Bifidobacterium bifidum*. LGS and LGSP promoted the growth of *Dubosiella newyorkensis*, but the LGSF had no effect.

**Conclusions:**

LGS exerts its anti-ALD effect in mice through regulating gut-liver axis, and its flavonoid and polysaccharide fractions, LGSF and LGSP, are responsible for its protective effect.

**Supplementary Information:**

The online version contains supplementary material available at 10.1186/s13020-024-01049-y.

## Introduction

Alcoholic liver disease (ALD) is a condition in which the liver is damaged by chronic alcoholism. It includes various liver abnormalities, from fatty liver to hepatitis, cirrhosis, and even liver cancer, making it a major factor in global illness and death [1, 2]. The proportion of alcohol consumers in China is about 20–40%, and the prevalence of ALD has reached about 10%, which has become a chronic disease in China [3].

Alcohol is absorbed mainly in the gastrointestinal tract and then distributed through the bloodstream in various organs and eventually metabolized in the liver. The metabolic processes involve two primary enzyme systems: alcohol dehydrogenase (ADH) and microsomal cytochrome P450 enzymes. ADH converts alcohol into acetaldehyde, an intermediary metabolite that is toxic, and acetaldehyde is further metabolized into acetic acid by acetaldehyde dehydrogenase (ALDH). Acetic acid is then converted into acetyl-CoA, which enters the tricarboxylic acid cycle. Ultimately, acetyl-CoA is broken down into water and carbon dioxide, which are expelled by respiration and urine [4, 5].

Alcohol and its metabolites can change the structure of intestinal microbiota and damage the gut mucosa and barrier, which allows an abundance of bacteria to enter the intestine, activates the immune response, and stimulates the liver to generate pro-inflammatory factors [6, 7]. As a result, the dysregulation of gut-liver axis is crucial for the progression of ALD. In 1998, Marshall introduced the gut-liver axis, which has since been demonstrated to significantly influence the pathogenesis and therapeutic approaches for various liver and intestinal disorders [8]. The gut-liver axis is a two-way communication process; due to the portal vein’s vascular access, the microbial community, metabolites and immune factors in the gut affect the liver function, and the bile and other substances secreted by the liver feed back to the intestine to regulate the intestinal environment and microbiota structure [9–11]. The gut-liver axis suggested a strong association between intestinal and liver physiological and pathological alterations. Recent focus has intensified on addressing ALD by rectifying disruptions in intestinal barrier and gut microbiota. Understanding the origins of gut microbiota shifts as well as the intestinal barrier function attributed to ALD is crucial for devising strategies to ameliorate the condition.

Presently, there are still no effective drugs authorized for the therapy of ALD, and abstinence from alcohol is considered as the best prevention and treatment strategy for ALD [12]. Most drugs including vitamin E, metformin, and statins are generally symptomatic treatments of ALD, and their therapeutic effects are not always satisfactory [13, 14]. Consequently, discoverying an efficient ALD therapy with few side effects is always needed.

Recently, natural medicine including Chinese herbal medicine (CHM) has garnered increasing research attention due to its multi-target, multi-pathway characteristics, as well as low toxicity and minimal side effects [15, 16]. For instance, silymarin derived from the milk thistle has long been used for hepatoprotection [17]. Liuweizhiji Gegen-Sangshen oral liquid (LGS) is derived from a Traditional Chinese medical formula that has been widely utilized in the prevention and treatment of ALD clinically [18, 19]. LGS consists of six Chinese medicinal herbs, including *Puerariae lobatae* radix, *Hoveniae* semen, *Imperatae* rhizoma, *Crataegi* fructus, *Mori* fructus and *Canarli* fructus [18]. It was reported that LGS dose-dependently mitigated ALD in rat models through improving ethanol metabolism [20]. Our previous work demonstrated that LGS contained a large number of flavonoids in LGS such as puerarin, dihydromyricetin, quercetin, vitexin, onion, etc. [18], some of which have been previously validated with potential therapeutic effect against ALD [21–23]. Moreover, LGS was enriched with polysaccharides, with a content of 17.94 ± 0.28 mg/mL [24]. It was found that LGS polysaccharides (LGSP) possessed remarkable anti-inflammatory and prebiotic activities in vitro [24]. It can be speculated that the flavonoid and polysaccharide fractions of LGS (LGSF and LGSP) resultant from manufacturing process are potentially the main effective constituents for ALD alleviation. However, till now whether and how the flavonoid and polysaccharide fractions of LGS contributes to the anti-ALD effect remains unclear.

As the dose-dependent therapeutic effect of LGS has been previously revealed [20], in this work, we hypothesize that LGS together with LGSF and LGSP fractions regulates the gut-liver axis and exerts an anti-ALD effect. An in vivo model of ALD using previously reported chronic and binge ethanol feeding method [25] was developed. Moreover, in vitro intestinal organoids and bacterial culture were used for the assessment of drug actions. The results will help understand the chemical basis of LGS for ALD alleviation as well as the underlying mechanisms.

## Materials and methods

### Chemicals and reagents

Lieber-DeCarli alcoholic liquid feed (#TP4020C) and control liquid diet (#TP4030D) was obtained from Trophic Animal Feed High-Tech Co., Ltd (Nantong, China). Food-grade ethanol was provided by Chengdu Kelong Chemical Co., Ltd (Chengdu, China). Meltadosine (MTDX) was purchased from Zhejiang Zhenyuan Pharmaceutical Co., Ltd (Shaoxing, China). Assay kits for determining ALDH, ADH, total cholesterol (TC), triglyceride (TG), low-density lipoprotein (LDL) and high-density lipoprotein (HDL) were purchased from Jiangsu Edison Biotechnology Co., Ltd (Jiangsu, China). Kits for aspartate transaminase (AST), alanine aminotransferase (ALT), tumor necrosis factor-α (TNF-α), interleukin-1β (IL-1β), interleukin-6 (IL-6), lipopolysaccharide (LPS) and glucagon-like peptide 1 (GLP-1) were supplied by Jiangsu Meibiao Biotechnology Co., Ltd (Jiangsu, China). Intesticult™ Organoid Growth Medium (IOGM) (mouse) was purchased from Stem Cell Technologies (Vancouver, Canada). Matrigel (#356231) was purchased from Corning Incorporated (New York, USA). TNF-α was purchased from Peprotech Biotechnology Co., Ltd (New Jersey, USA).

*Bacillus coagulans* (CICC 20138), *Bifidobacterium bifidum* (CICC 6071), *Bifidobacterium adolescentis* (CICC 6070), and *Lactobacillus* (CICC 6269) were obtained from the China Center of Industrial Culture Collection (CICC, Beijing, China). *Dubosiella newyorkensis* (ATCC TSD-64) and *Bacteroides sartorii* (bio-107029) were obtained from Beijing BioBowei Biotechnology Co., Ltd (Beijing, China). MRS medium (Shandong Tuopu Biol-engineering, Shandong, China) was used to cultivate *Lactobacillus*, *B. coagulans*, and *B. sartorii*, while CM0233 medium (Qingdao Hi-tech Industrial Park Hope Bio-technology, Shandong, China) was used to cultivate *B. bifidum*, *B. adolescentis* and *D. newyorkensis*. The above bacteria were cultured at a constant temperature of 37 °C in an anaerobic environment. In addition, the corresponding sugar-free medium was prepared for use.

### Preparation and standardization of LGSP and LGSF fractions

LGS was provided by Sichuan Tongyou Life Health Technology Co., Ltd (Sichuan, China). Voucher specimens (Batch Number: 230110) were stored at the School of Pharmacy, Southwest Medical University, Luzhou, China. Standardization of different batches of LGS, regarding both the small-molecule compounds and the macro-molecule polysaccharides, was performed in our previous works [18, 24].

The polysaccharide fraction LGSP were extracted using the 95% alcohol precipitation method as we previously reported [24]. Specifically, a thermostable α-amylase was added into LGS at a concentration of 10 U/mL and incubated at 70 °C for 8 h. Glycase enzymes were added at a dosage of 10 U/mL to remove starch after cold. After 2 h of inactivation at 59 °C for 2 h, and finally heated to 95 °C for 30 min. Subsequently, 4 volumes of 95% (*v*/*v*) alcohol were added to precipitate the crude polysaccharides which was washed repeatedly with 95% alcohol, then dissolved in distilled water and freeze-dried, and stored at 4 °C for use. Quality of LGSP was evaluated as we previously reported [24]. The extraction rate of LGSP was 17.83 mg/mL LGS, and the total polysaccharide content (purity) in LGSP was 82.64%. The prepared LGSP contained 21.23% total uronic acid and 2.78% total protein, and were composed of 8 monosaccharide components: mannose (Man), glucose (Glc), galactose (Gal), xylose (Xyl), rhamnose (Rha), arabinose (Ara), glucuronic acid (GlcA), and galacturonic acid (GalA).

For preparation of the flavonoid fraction LGSF, AB-8 resin purchased from Tianjin Bohong Resin Technology Co., Ltd (Tianjin, China) was used. The sample loading volume was 200 mL and the ratio of AB-8 resin packing volume to sample loading was 1:1. Firstly, 4–5 bed volumes (BVs) of distilled water was used to elute impurities until the eluate was colorless; Then, 3 BVs of 70% ethanol was used; Finally, vacuum-rotary evaporation procedure was applied to remove the ethanol, followed by drying in a vacuum freeze dryer. The sodium nitrite-aluminum nitrate method was used to determine the flavonoid content in LGSF, and the chemical constituents in LGSF were assessed by a HPLC method. As a result, the yield of LGSF was 46 mg/mL LGS, and total flavonoid content in LGSF was determined as 63.3%. LGSF contained mainly the puerarin, 3′-hydroxypuerarin, puerarin apioside, genistin, onion and daidzein (Fig. S1).

### Animals

A total of 60 male 6-week-old C57BL/6J mice of specific-pathogen-free grade were purchased from Beijing Huafukang Biotechnology Co., Ltd (Beijing, China). All mice were kept at the Animal Experiment Center (SPF level) of Southwest Medical University, with a temperature of 23 ± 1 °C and a humidity of 40–60%. Every 12 h, the day and night were alternated, and the mice were subjected to acclimatization for 1 week before experiment with free access to drinking water and standard diet. All experiments were complied with the guidelines for the care and use of laboratory animals set forth by the National Institutes of Health, and were approved by the Ethics Committee of Southwest Medical University (Approval No. 20221026-022).

### Establishment of ALD mouse model and drug treatment

**Fig. 1:**
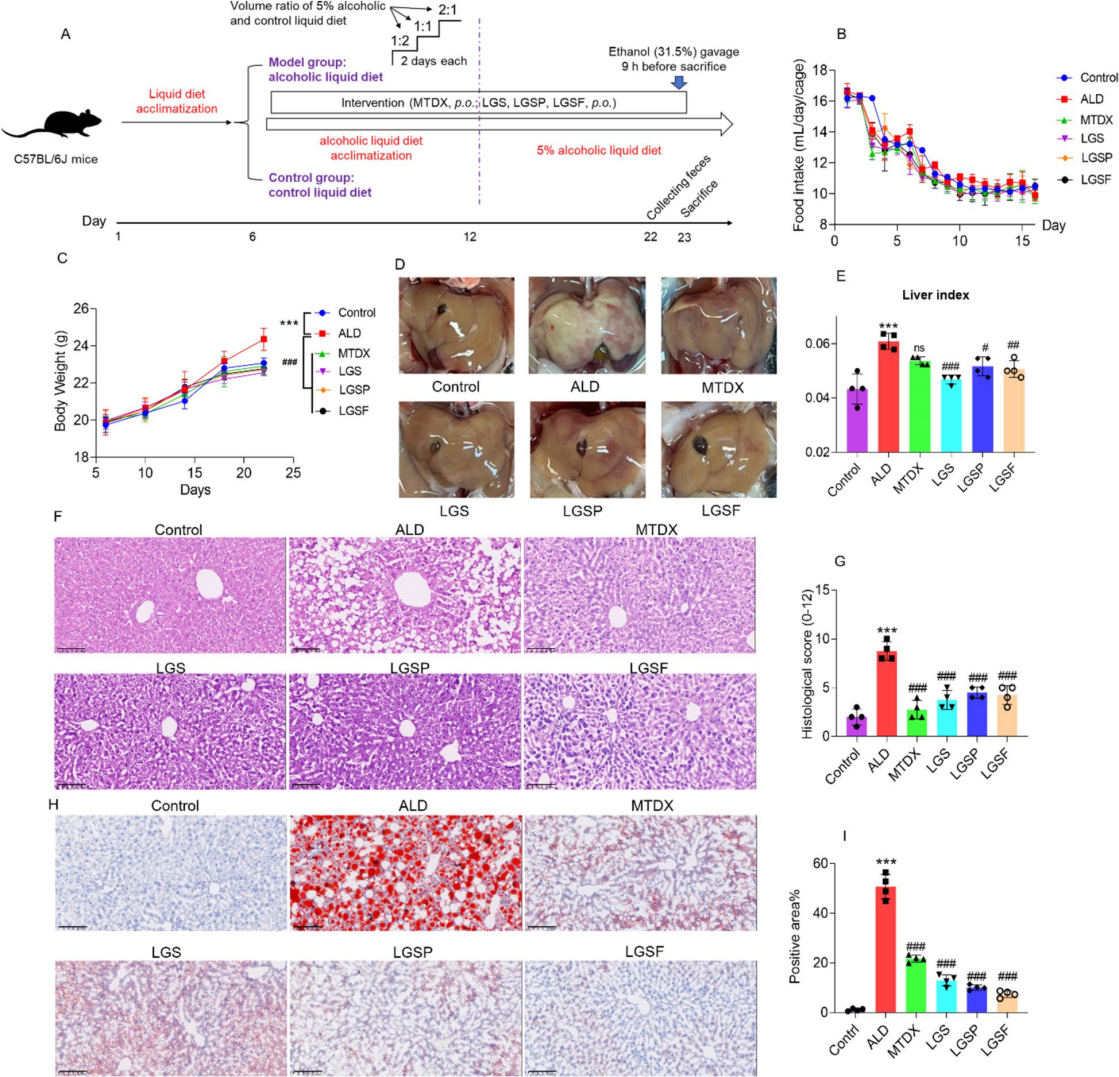
LGS and its active fractions (LGSP and LGSF) attenuated alcohol-induced hepatic injury. **A** Experimental design. **B** Food intake curve of mice. **C** Body weight change curve of mice. **D** Representative morphological images of the liver. **E** Liver index. **F** H&E-stained sections of mouse liver. **G** Pathologic scoring of H&E sections of the liver. **H** Oil red O staining of liver sections. **I** Positive area for oil red O staining. ^#^ *P* < 0.05, ^##^ *P* < 0.01, ^###^ *P* < 0.001, vs. ALD group; * *P* < 0.05, ** *P* < 0.01 and *** *P* < 0.001, vs. control group; one-way ANOVA with a post hoc Tukey test

The establishment of ALD mouse model was conducted according to the previously reported chronic and binge ethanol feeding method, the NIAAA method [25]. In brief, all mice were adaptively fed with the control liquid diet for 5 days, followed by a 6-day (*v*/*v*) graded alcohol transition feeding period by gradually escalating the alcohol concentration in diet (by mixing the Lieber-DeCarli alcohol-free high-fat liquid diet and 5% alcoholic liquid diet at volume ratios of 2:1, 1:1, and 1:2, respectively; each for 2 days). Subsequently, mice were given 5% alcohol liquid diet continually for another 10 days, and were orally gavaged with a high dose of alcohol at 31.5% (*v*/*v*) at 9 h before the end of experiment. Mice in the control group were fed with a control liquid diet with normal fat level, and were orally gavaged with isocaloric maltodextrin (45%, w/v). All groups received with equal calorie intake. The detailed modeling process is shown Fig. 1A.

Animals are randomly divided into six groups (*n* = 10 per group): Control group, ALD group, MTDX group (positive control), LGS group, LGSP group and LGSF group. The drug administration was initiated at the beginning of the graded alcohol transition feeding period (Fig. 1A), and the oral dosages were 200 mg/kg once daily for metadoxine, 2500 mg/kg (equivalent to 12.5 mL LGS per kg body weight; equivalent to human daily dose) once daily for LGS, 270 mg/kg (equivalent to LGS dose) once daily for LGSP, 900 mg/kg (equivalent to LGS dose) once daily for LGSF, and the identical volume of regular saline was administered to the ALD group and the Control group.

Fecal specimens were collected from mice at 2 days before the end of experiment, which were stored at −80 °C. After anaesthetization of mice, blood was collected and further processed to obtain serum. After sacrifice of mice, liver and intestinal tissues were collected for subsequent analysis.

### Biochemical analysis

The levels of serum TG, TC, LDL, HDL and LPS were measured using the corresponding kits according to the instructions of the manufacturers. Furthermore, the contents of AST, ALT, IL-1β, IL-6, TNF-α, and GLP-1 in mouse serum were analyzed using ELISA kits as per the manufacturer’s instructions.

The activity of ADH and ALDH enzymes was determined by corresponding kits. In brief, a 20 μL serum sample was used for analysis. ADH or ALDH will utilize specific substrate leading to a proportional color development. The activity of ADH or ALDH was quantified calorimetrically at 450 nm.

### H&E staining

Liver, colon and ileum tissues were preserved in 4% paraformaldehyde followed by paraffin embedding. Hematoxylin and eosin (H&E) staining was carried out using a previously established protocol [26, 27]. Scanning analysis was conducted using a digital pathology slide scanner (KF-PRO-002, Zhejiang, China). Liver histopathology was scored in terms of inflammatory infiltration, hepatocyte ballooning, steatosis, and hepatic fibrosis [26, 27], with a total score of 12 points and a range of 0–3 points for each indicator. Colon and ileum injuries were analyzed for epithelial injury (0–4), inflammatory infiltrate (0–4), and mucosal abnormality (0–4), and their histologic scores were calculated based on the sum of the scores [26, 27].

### Oil red O staining

Liver tissue samples were frozen using liquid nitrogen, cut into thin slices, and stained with oil red O solution (Servicebio, Wuhan, China). The samples were then immersed in 60% isopropanol (Servicebio, Wuhan, China) for background differentiation, and counterstained with hematoxylin dye (Servicebio, Wuhan, China). The staining effect was observed and analyzed using a digital pathology slide scanner (KF-PRO-002, Zhejiang, China).

### TUNEL assay

Sections embedded in paraffin were deparaffinized and then incubated at 37 °C in an incubator with proteinase K (Servicebio, Wuhan, China) for 22 min. Subsequent steps were carried out following the instructions of the TUNEL kit (Servicebio, Wuhan, China), images were observed and captured using a Nikon Eclipse C microscope (Tokyo, Japan).

### Immunohistochemistry (IHC) analysis

The paraffin sections were deparaffinized to water, followed by antigen retrieval using citric acid solution (pH 6.0) (Servicebio, Wuhan, China), and blocking of endogenous peroxidase with 3% hydrogen peroxide (Servicebio, Wuhan, China) was performed on the sections. After blocking with 3% BSA, the primary antibody (zona occludens 1 (ZO-1) or occludin, Servicebio, Wuhan, China) was added overnight, then the secondary antibody was incubated for 50 min. After adding the DAB stain (Servicebio, Wuhan, China) to develop the color, the nuclei were finally restained with hematoxylin (Servicebio, Wuhan, China). Images were viewed and recorded using a Nikon Eclipse C microscope (Tokyo, Japan).

### Immunofluorescence (IF) analysis

Paraffin sections were deparaffinized to water, then antigen retrieval solution was added for repair, and BSA (Servicebio, Wuhan, China) was added for blocking after repair. After incubating with the primary antibody (Lgr5, Muc2, and Lyz1) overnight, the corresponding secondary antibody was added and incubated in the dark for 50 min. The nuclei were counterstained with DAPI stain (Servicebio, Wuhan, China), and finally the antifluorescence quencher (Servicebio, Wuhan, China) was added to mount, and the staining results were viewed and recorded using an Olympus BX63 fluorescence microscope (Tokyo, Japan).

### 16S rRNA analysis

Fecal DNA extraction, PCR amplification and amplicon purification were performed according to the previous method [28, 29]. According to the standard procedures, Majorbio Bio-Pharm Technology Co. Ltd (Shanghai, China) carried out the MiSeq sequencing on an Illumina MiSeq platform [30]. The microbial analysis was performed at the free on-line Majorbio Cloud Platform (http://www.majorbio.com) [30]. Dilution curves and α diversity were analyzed with Mothur v1.30.1 and β diversity was analyzed with QIIME. The Bray–Curtis algorithm was used to analyze principal coordinate analysis (PCoA) and to observe the differences among groups. The coupling of linear discriminant analysis (LDA) to effect sizes (LEfSe) was implemented using the LEfSe program.

### Organoid culture and treatments

The small intestine was amputated at the proximal stomach end of the mouse, with the mesentery removed, followed by rinsing 5 times with PBS, and cutting into 3–5 mm segments. The intestinal segments were added into 20 mL of PBS containing 5 mM EDTA and shaken on ice for about 15 min at 40 rpm. The isolated crypts were resuspended in a mixture of IOGM and Matrigel at a volume ratio of 3:7, with 2000 crypt cells per 50 μL mixture. The crypt suspension was seeded into a pre-warmed 24-well plate, and after the Matrigel solidified, pre-warmed IOGM 450 μL was added to each well and cultured at 37 °C in 5% CO_2_.

The morphology of the organoids was checked under a light microscope during the culture process. After the organoids germinated, the model group received 500 ng/mL of TNF-α for 24 h, while the control group received an equivalent volume of medium. After the TNF-α challenge, organoids were treated for 24 h with LGS, LGSP, and LGSF at 5 μg/mL, respectively. At the end of experiment, the organoids were collected, embedded with agar, and then subjected for H&E staining, EdU staining, TUNEL staining and IF analysis.

### EdU staining

The paraffin sections were deparaffinized, and treated with PBS and osmotic agent, with the EdU staining reaction solution (Servicebio, Wuhan, China) added for 30 min in the dark. Then the sections were treated with DAPI staining solution (Servicebio, Wuhan, China) under the condition of avoiding light, washed with PBS and added with fluorescence quencher (Servicebio, Wuhan, China) for covering, and finally the image acquisition was performed under the IKON ECLIPSE C1 fluorescence microscope (Tokyo, Japan).

### Measurement of bacterial growth

To assess the impact of LGS, LGSP, and LGSF on specific bacterial growth, glucose-free culture medium was used. LGS at a concentration of 4000 mg/L, LGSP at 1000 mg/L and LGSF at 600 mg/L were used as the main carbon sources in culture medium. The selection of concentration of LGSP was according to our previous report [24], and the equivalent concentration for LGS and LGSF was further calculated based on the relative yields of LGSP and LGSF. A concentration of 1000 mg/L glucose was used as a positive control. Sugar-free medium serves as a blank control in the experiment. Bacterial growth was measured at designated time points, respectively. The optical density (OD) of the bacterial solution at 600 nm was measured using a spectrophotometer, and bacterial pellet was photographically recorded at each time point.

### Statistical analysis

Graphpad Prism 8.0 software (USA) was used for statistical analysis. One-way ANOVA with a post hoc Tukey test was applied for comparison of multiple groups. A *p* value less than 0.05 was considered as statistically significant.

## Results

### LGS, LGSP, and LGSF attenuated ALD abnormality in mice

The food intake of mice is shown in Fig. 1B, and the food intake in each group was comparable. At the end of experiment, the weight of mice in ALD group was slightly higher than that in the control group and other groups (Fig. 1C). Morphological observation showed that the liver in ALD mice was apparently in a whiter color compared to control mice, indicating the fat accumulation (Fig. 1D). After treatment by MDTX, LGS, LGSP and LGSF, the liver of mice became ruddy (Fig. 1D). Furthermore, as shown in Fig. 1E, compared with the control group, the liver index was significantly increased in the ALD group (*p* < 0.001), which was alleviated by treatment of LGS, LGSP, and LGSF, with the most pronounced remission effect by LGS.

Histological analysis of liver tissue showed that excessive alcohol intake could lead to the appearance of a large number of lipid vacuoles in the liver and cause the destruction of hepatocyte structure, while the MTDX, LGS, LGSF and LGSP treatment apparently mitigated these histological changes (Fig. 1F, G). In addition, the protective effect of MTDX, LGS, LGSP, and LGSF on alcohol-induced hepatic steatosis was verified by examining liver fat accumulation using oil red O staining. As shown in Fig. 1H and I, the ALD group exhibited severe fat accumulation (red), which was attenuated in the MTDX, LGS, LGSP, and LGSF groups.

Overall, LGS and its LGSP and LGSF fractions had a protective effect in ALD mice.

### LGS, LGSP, and LGSF protected against ALD in mice through alleviating hepatic injury and inflammation, improving dyslipidemia and promoting ethanol metabolism

**Fig. 2:**
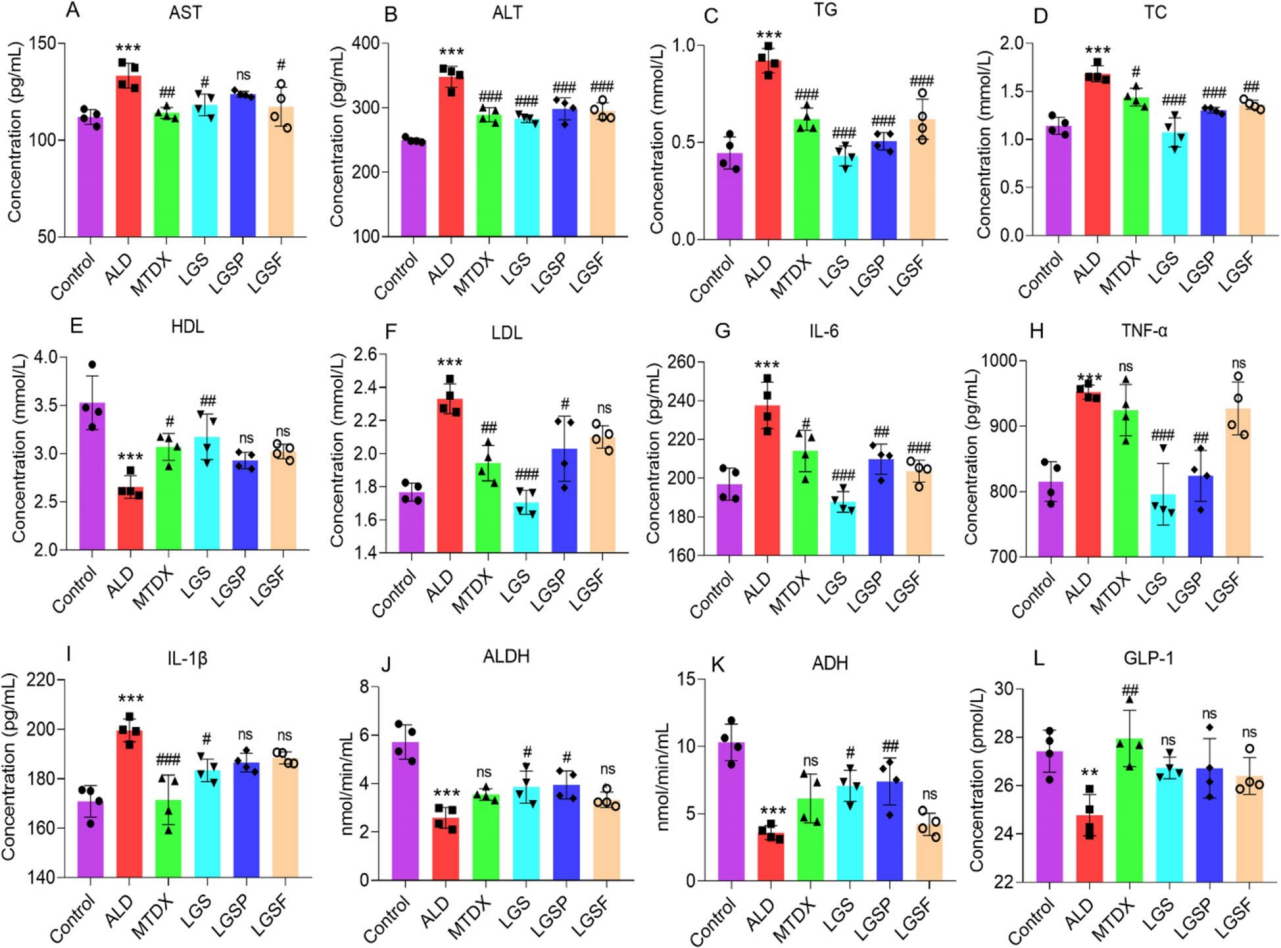
LGS and its active fractions (LGSP and LGSF) alleviated hyperlipidemia and inflammation, and improved ethanol metabolism in ALD mice. **A** Serum level of AST; **B** serum level of ALT; **C** serum level of TG; **D** serum level of TC; **E** serum level of HDL; **F** serum level of LDL; **G** serum level of IL-6; **H** serum level of TNF-α; **I** serum level of IL-1β; **J** enzymatic activity of ALDH; **K** enzymatic activity of ADH; **L** serum level of GLP-1. ^#^ *P* < 0.05, ^##^ *P* < 0.01, ^###^ *P* < 0.001 vs. ALD group; * *P* < 0.05, ** *P* < 0.01 and *** *P* < 0.001 vs. control group; one-way ANOVA with a post hoc Tukey test

Serum AST and ALT levels are sensitive indicators of liver function [31]. Alcohol intake has a significant effect on liver-specific enzymes, including AST and ALT. As shown in Fig. 2A and B, compared to control group, there was a significant elevation of AST and ALT in the ALD group, indicating that the abnormal liver function. Both MTDX and LGS treatment similarly and significantly reduced the alcohol-induced AST and ALT elevation. LGSP had a significant remission effect on ALT level, while LGSF showed alleviating effect on both AST and ALT levels.

Alcohol affects fat metabolism in the liver, causing abnormalities in processes such as fat synthesis, transport, and oxidation, which ultimately leads to fat accumulation in the liver [32]. The levels of TC, TG, LDL and HDL serve as sensitive markers for assessing liver lipid metabolism disorders. The results (Fig. 2C–F) demonstrated that MTDX and LGS significantly mitigated the ethanol-induced increase of TC, TG and LDL, and decrease of HDL. Both LGFP and LGSF significantly reduced serum TC and TG levels in ALD mice, while they had no effect on HDL. LGSP reduced LDL while LGSF did not.

Furthermore, LGS treatment significantly reduced the ethanol-induced increase of proinflammatory factors IL-6, TNF-α and IL-1β (Fig. 2G–I). MTDX only had the remission effect on IL-6 and IL-1β. LGSP decreased the serum levels of IL-6 and TNF-α, while LGSF only had an effect on IL-6 but not TNF-α. Both LGSP and LGSF had no effect on IL-1β.

Alcohol-induced liver damage might be linked to the activity of the main catalytic enzymes ADH and ALDH in the liver [33]. In this study, the enzymatic activities of ALDH and ADH were significantly lower in ALD group, and the LGS and LGSP treatment partially recovered the enzyme activities (Fig. 2J, K). Both MTDX and LGSF had no mitigating effect on activities of ALDH or ADH. In addition, although LGS, LGSP and LGSF groups showed a trend of increase on ethanol-induced serum GLP-1 level, no statistical difference was found (Fig. 2L).

Based on above results, LGS had a remarkable effect on mitigating dyslipidemia, hepatic injury and inflammation, and improving ethanol metabolism. Regarding most of the detected indices, the protective effect of LGS was comparable to that of MTDX, while LGSP or LGSF displayed a relatively smaller effect.

### LGS, LGSP, and LGSF mitigated intestinal damage and maintained barrier function in ALD mice

Alcohol abuse can cause damage to the intestinal mucosa, leading to epithelial cell damage and necrosis, thereby compromising mucosal barrier function [34]. Therefore, we further explored the impact of LGS, LGSP, and LGSF on intestinal repair.

**Fig. 3:**
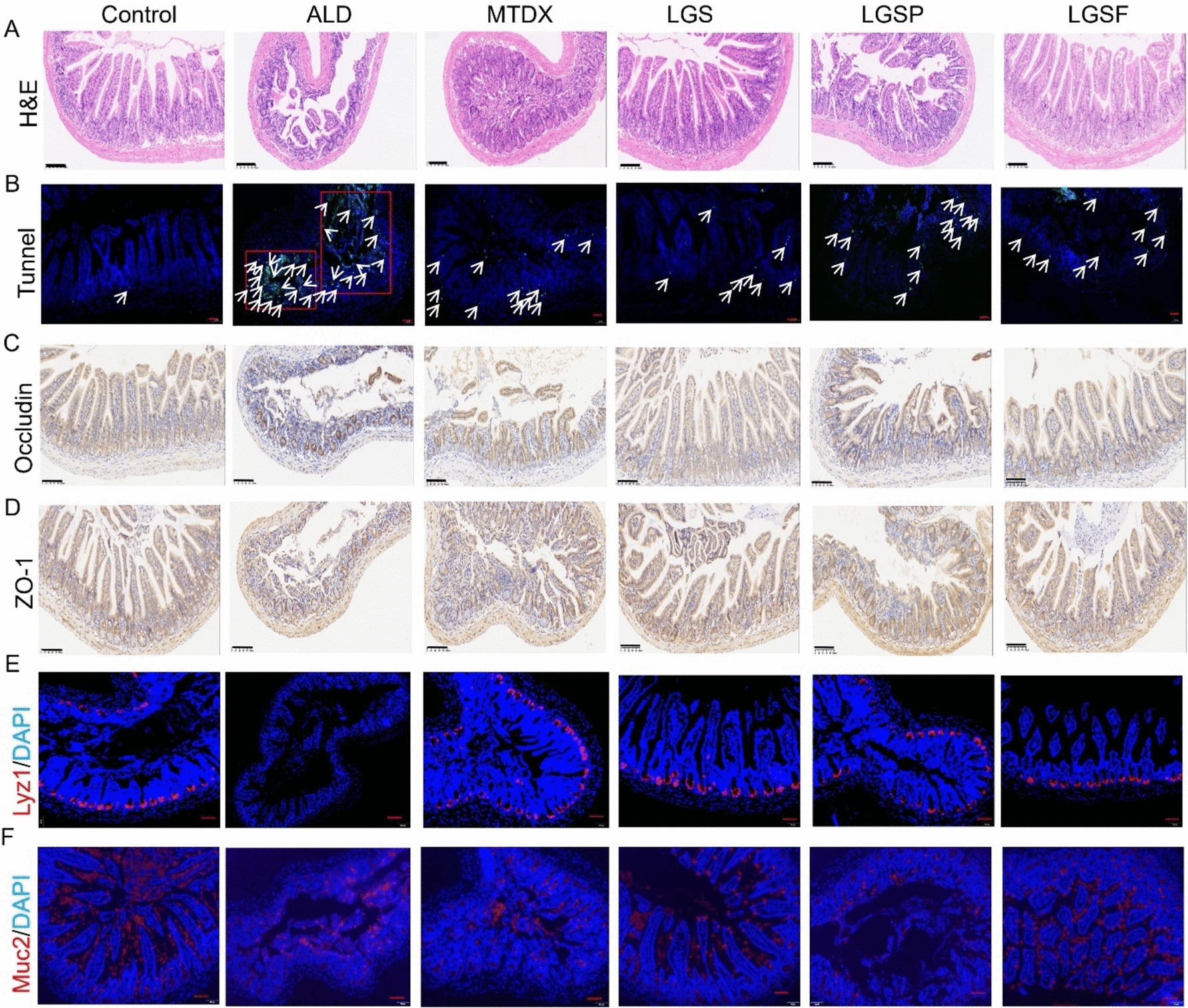
LGS and its active fractions (LGSP and LGSF) mitigated intestinal injury, increased the expression of tight conjunction proteins, and promoted intestinal epithelial proliferation in ALD mice. **A** H&E staining of ileum. **B** TUNEL-stained ileum sections. Arrows indicate apoptotic cells. **C** The expression of occludin protein in ileum. **D** The expression of ZO-1 protein in ileum. **E** Immunofluorescence staining of Lyz1^+^ cells in ileum; red, Lyz1 positive staining; blue, DAPI staining. **F** Immunofluorescence staining of Muc2^+^ cells in ileum; red, Muc2 positive staining; blue, DAPI staining. Quantitative analysis of histopathological scores, TUNEL-positive cells, occludin or ZO-1 positively stained area, and number of Lyz1^+^ and Muc2^+^ cells are displayed in the supplementary file (Figs. S3, S7)

As shown in Figs. 3A and S2A, H&E staining analysis of the ileum and colon showed that, compared with the control group, the ALD group showed inflammatory invasion, mucosal thickening and shedding, decreased goblet cells, shortened glandular tracts, and/or irregular arrangement of ileum villi, however, after treatment with LGS, LGSP, and LGSF, the extent of gut damage was significantly reduced, where the inflammatory cell infiltration was reduced, the intestinal epithelium was more intact, and the crypt structure was improved. According to the histopathological score, compared to the ALD group, the treatment groups’ histopathological scores were lower (Fig. S3A, B). In contrast, the positive drug MTDX had no protective effect on epithelial damage.

Additionally, we performed TUNEL apoptosis assays on the ileum (Figs. 3B, S3C) and colon (Figs. S2B, S3D), and the green stained cells represent the apoptotic cells. By counting the apoptotic cells, it was found that apoptotic cells in the ileum and colon of mice in the LGS, LGSP, and LGSF group were considerably lower than that in the ALD group.

To analyze the gut permeability, we determined the serum level of LPS. LPS, as a large molecule metabolite of bacteria that cannot readily move across gut barrier, has been used as a marker for gut permeability [26, 27]. The result showed that the serum level of LPS in LGS, LGSP, and LGSF treatment group was significantly decreased compared to the ALD group (Fig. S4). This suggests that LGS, LGSP, and LGSF presumably improved gut permeability.

The integrity of the intestinal barrier depends on tight junction proteins such as occludin, ZO-1, and other associated proteins [30]. ZO-1 and occludin play a crucial role in maintaining the integrity and permeability of the intestinal mucosa. The absence of ZO-1 and occludin increases intestinal permeability, resulting in the invasion of bacteria and viruses, which in turn affects the protective effect of the intestinal mucosa [35, 36]. Therefore, to further validate the gut barrier function, IHC was performed to further assess the levels of ZO-1 and occludin in both the ileum and colon. As shown in Figs. 3C, D, S3E–H and S5, there was an evident deletion of ZO-1 and occludin in the ileum and colon tissues of the ALD group, while in the LGS, LGSP, and LGSF treatment group, compared to the ALD group, the rates of positive expression for ZO-1 and occludin were noticeably higher. The expression of occludin in colon tissue did not show a significant difference among groups (Figs. S3F, S5A). To further elucidate the contributions of LGS, LGSP, and LGSF in preserving intestinal mucosa, we detected the expression of Paneth cell marker lyz1 and goblet cell marker muc2 in the ileum and colon by IF analysis [37]. As shown in Figs. 3E, F, S6 and S7, in ileum and colon, the number for Lyz1^+^ and Muc2^+^ cells (red) in LGS, LGSP, and LGSF groups were higher than those of the ALD group.

Overall, the above findings suggest that the use of LGS, LGSP, and LGSP inhibits intestinal damage and improves intestinal barrier function in the mouse model of ALD.

### LGS, LGSP, and LGSF promoted intestinal epithelial repair in TNF-α-induced intestinal organoids

**Fig. 4:**
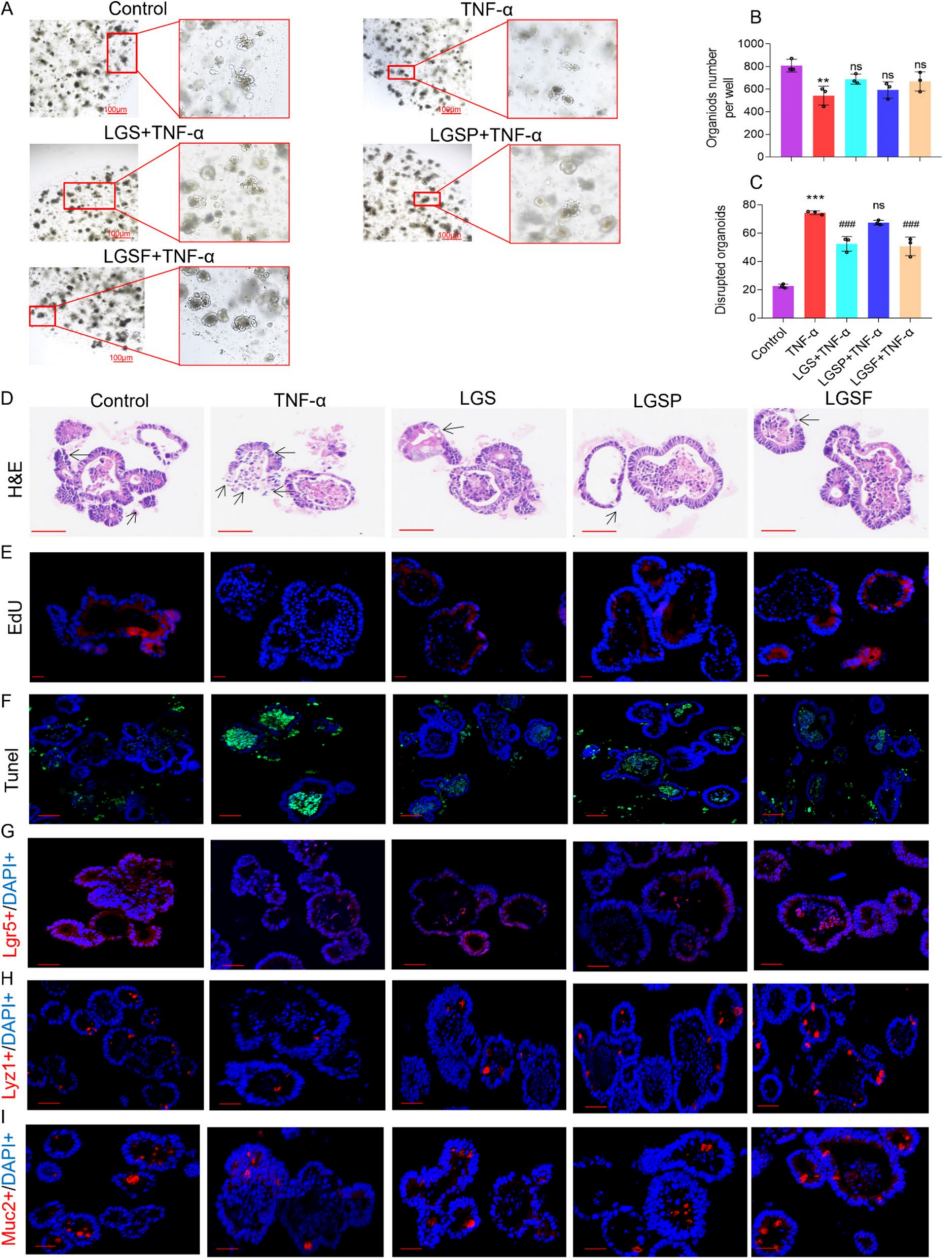
LGS and its active fractions (LGSP and LGSF) protected against TNF-α-induced intestinal organoid injury in vitro through promoting epithelial cell differentiation and regeneration. **A** Intestinal organoid image under light microscopy. Scale bar = 100 μm. **B** Number of total organoids. **C** Number of injured organoids. **D** H&E-stained organoids. Arrow shows bud loss or reduced surface area. Scale bar = 50 μm. **E** EdU-stained organoids; scale bar = 20 μm. **F** TUNEL stained organoid sections; scale bar = 50 μm. **G** Lgr5 staining; red, Lgr5 positive staining; blue, DAPI staining; scale bar = 50 μm. **H** Lyz1 staining; red, Lyz1 positive staining; blue, DAPI staining; scale bar = 50 μm. **I** Muc2 staining; red, Muc2 positive staining; blue, DAPI staining; scale bar = 50 μm. Quantitative analysis of EdU^+^ and TUNEL^+^ immunofluorescence, and the number of Lgr5^+^, Lyz1^+^ and Muc2^+^ cells are displayed in the supplementary file (Fig. S8). ^#^ *P* < 0.05, ^##^ *P* < 0.01, ^###^ *P* < 0.001 vs. ALD group. * *P* < 0.05, ** *P* < 0.01 and *** *P* < 0.001 vs. control group; one-way ANOVA with a post hoc Tukey test

Intestinal organoids are three-dimensional cellular models that encompass various types of functional intestinal epithelial cells, such as epithelial cells, goblet cells, and Paneth cells [38]. In view of this, we used TNF-α (500 ng/mL) to induce 24 h to construct an intestinal organoid inflammation model [39]. In the TNF-α group, the morphology of the organoids showed a reduction in surface area, blackening and loss of part of the bud morphology; in contrast, the intestinal organoids in the control group were morphologically normal (Fig. 4A). The results demonstrated that the inflammatory injury model was well established. The number of intestinal organoids was counted by microscopic observation. There was no obvious distinction in the number of organoids among groups (Fig. 4B), while compared to TNF-α group, the calculated impairment rates showed a significant decrease in the LGS and LGSF groups, with a decrease trend in the LGSP group, but there was no statistical difference (Fig. 4C).

H&E staining (Fig. 4D) showed that the organoids in the control group are morphologically intact and the cells are tightly arranged, while the TNF-α group showed bud loss or reduced surface area, compared with the TNF-α group. After the treatment of LGS, LGSP, and LGSF, the germ emergence length and proportion of organoids were significantly higher, and the surface area was larger. In addition, EdU staining and TUNEL assay were applied to detect cell proliferation and apoptosis in each group. It was further found that after LGS, LGSP, and LGSF treatment, the positive area (red) was significantly increased (Figs. 4E, S8A), which indicated the onset of regeneration. Meanwhile, LGS, LGSP, LGSF also reduced the apoptosis of intestinal epithelial cells caused by TNF-α (Figs. 4F, S8B).

Notably, we also detected organoid epithelial cell levels in each group by IF staining of epithelial cell markers such as Lgr5, Lyz1, and Muc2. It was found that LGS, LGSP, and LGSF treatment up-regulated the TNF-α-mediated decrease of Lgr5 (Figs. 4G, S8C), Lyz1 (Figs. 4H, S8D), and Muc2 (Figs. 4I, S8E) expressions.

These findings suggest that LGS, LGSP, and LGSF can enhance the proliferation of intestinal epithelial cells, reduce apoptosis, and thus promote the recovery of intestinal function.

### LGS, LGSP, and LGSF alleviated gut dysbiosis in ALD mice

Gut microbiota plays a crucial role in maintaining gut barrier function, and has been suggested tightly associated with ALD development. Thus, we further assessed the impact of LGS, LGSP, and LGSF on gut microbiota in ALD mice.

**Fig. 5:**
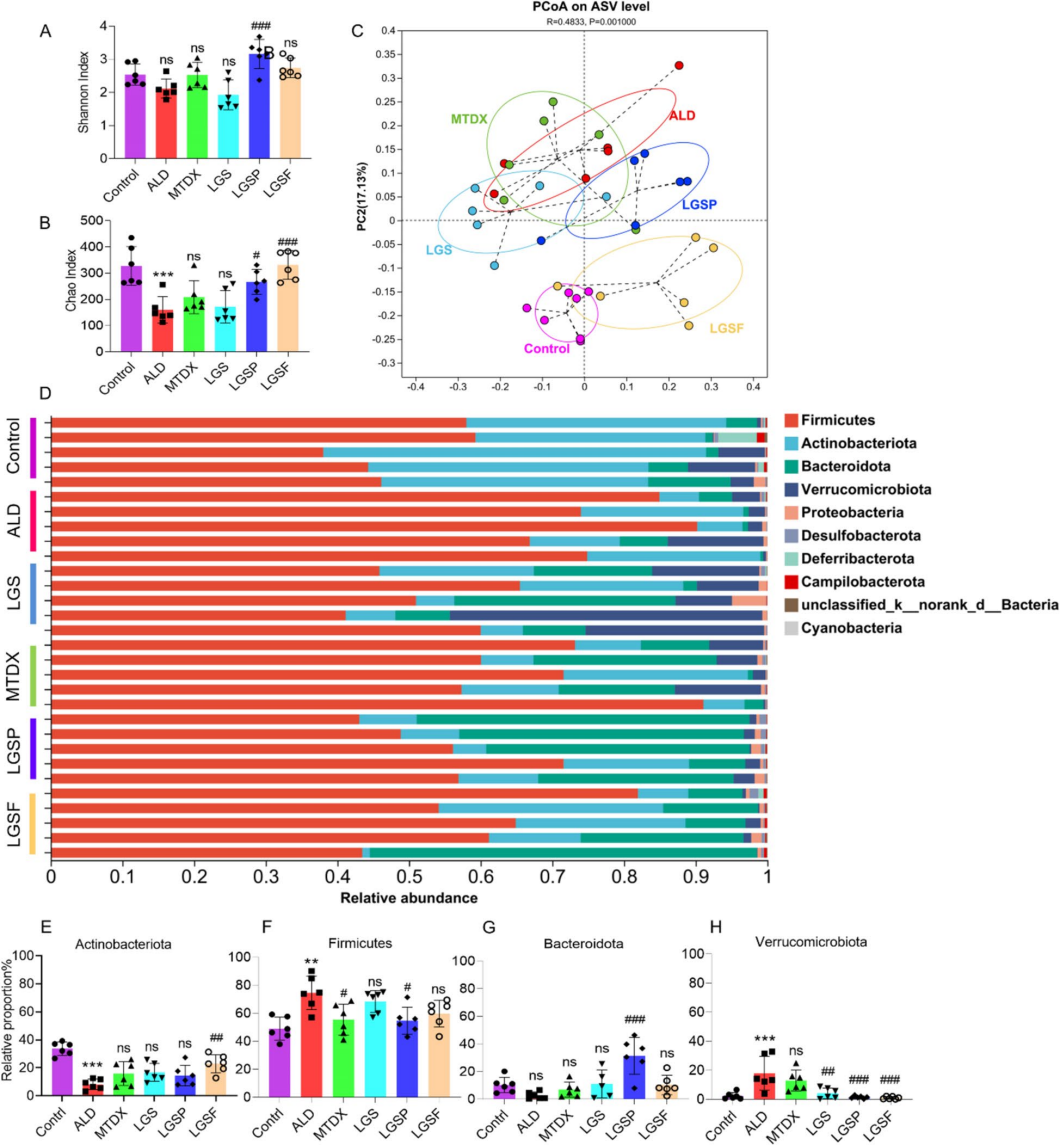
LGS and its active fractions (LGSP and LGSF) altered gut microbial structure in ALD mice. **A** Shannon index. **B** Chao1 index. **C** PCoA analysis based on ASV level. **D** Percent of community abundance on phylum level. **E** Abundance of Actinobacteriota. **F** Abundance of Firmicutes. **G** Abundance of Bacteroidota. **H** Abundance of Verrucomicrobiota. ^#^ *P* < 0.05, ^##^ *P* < 0.01, ^###^ *P* < 0.001 vs. ALD group; * *P* < 0.05, ** *P* < 0.01 and *** *P* < 0.001 vs. control group; one-way ANOVA with a post hoc Tukey test

The α diversity of microorganisms was assessed using the Shannon and Chao1 indices (Fig. 5A, B). In comparison to the control group, alcoholic diet did not significantly alter the diversity of fecal microbes in mice, as the Shannon index was not changed. The values of Shannon index in the LGSP group were much higher than those of the ALD group, indicating LGSP increased microbial diversity. LGSP and LGSF also recovered ALD-mediated reduction of bacterial richness (Chao1 index).

Beta diversity can be used to intuitively compare the structure of microbe communities and analyze the differences in microbe communities between groups. PCoA analysis based on ASV level (Fig. 5C) showed that ALD mice were far separated from the control mice, showing vast distinct microbial structures. The MTDX group was placed close to the ALD group, suggesting that MTDX treatment had minimal effect on microbial compositions. However, after treatment of LGS, LGSP and LGSF, it is observed that the microbial structure was altered in a way moving towards the control group. The LGS and ALD groups were relatively close in distance, and LGSP and LGSF groups were significantly distinguished from the ALD group, with the LGSF group was the most obvious which was close to the control group. The above results suggested that the intervention of LGS, LGSP, and LGSF affected the structure of the microbiota to various extents, and LGSF caused the greatest changes in the structure of the microbiome.

Subsequently, the microbial composition was assessed. At the phylum level (Fig. 5D), Actinobacteriota (Fig. 5E), Firmicutes (Fig. 5F), Bacteroidetes (Fig. 5G), and Verrucomicrobiota (Fig. 5H) were the dominant bacterial groups, constituting over 90% of the total sequences. The proportion of Actinobacteriota was decreased significantly from 33.68 ± 4.3% in the control group to 8.15 ± 3.44% in ALD group, which was further increased to 15.88 ± 7.77% (MTDX group), 16.78 ± 5.82% (LGS group), 14.45 ± 6.69% (LGSP group), 22.96 ± 5.9% (LGSF group), with statistical difference observed for LGSF group (Fig. 5E). The proportion of Firmicutes increased significantly from 49.04 ± 7.43% in the control group to 74.65 ± 10.91% in the ALD group, and a significant decrease was observed after treatment of MTDX and LGSP (Fig. 5F). In addition, the proportion of Bacteroidota was increased specifically in the LGSP group (Fig. 5G). Compared with the control group (2.34 ± 2.14%), the percentage of Verrucomicrobiota was increased to 17.97 ± 10.56% in the ALD group, of which showed a significant decrease in the LGS (4.2 ± 3.3%), LGSP (1.48 ± 0.84%), and LGSF (1.03 ± 0.78%) groups (Fig. 5H).

**Fig. 6:**
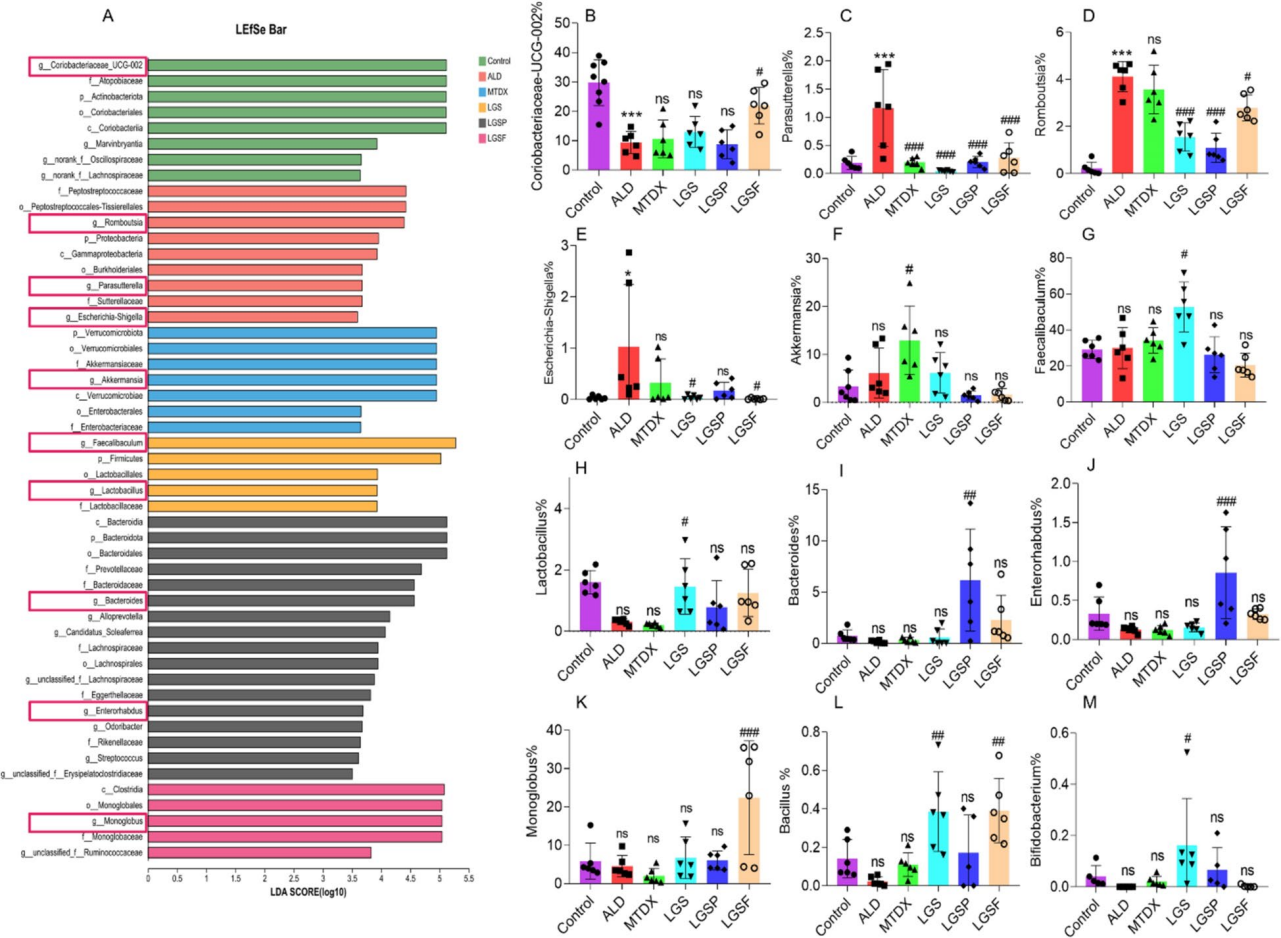
LGS and its active fractions (LGSP and LGSF) mediated specific microbial changes in ALD mice. **A** LEfSe analysis of the microbial community in each group. The significantly altered microbial genera included the following: **B** *Coriobacteriaceae-UCG-002*; **C** *Parasutterella*; **D** *Romboutsia*; **E** *Escherichia-shigella*; **F** *Akkermansia*; **G** *Faecalibaculum*; **H** *Lactobacillus*; **I** *Bacteroides*; **J** *Enterorhabdus*; **K** *Monoglobus*; **L** *Bacillus*; **M** *Bifidobacterium*. ^#^ *P* < 0.05, ^##^ *P* < 0.01, ^###^ *P* < 0.001 vs. ALD group; * *P* < 0.05, ** *P* < 0.01 and *** *P* < 0.001 vs. control group; one-way ANOVA with a post hoc Tukey test

In order to find the specific major microbes between the groups, a linear discriminant analysis (LDA) effect size (LEfSe) analysis was performed (Fig. 6A). The results showed that certain bacterial genera were enriched in each group (Fig. 6A): *Coriobacteriaceae-UCG-002* in the control group; *Parasutterella*, *Romboutsia* and *Escherichia-shigella* in the ALD group; *Faecalibaculum* and *Lactobacillus* in the LGS group; *Enterorhabdus*, *Alloprevotella*, and *Bacteroides* in the LGSP group; and *Monoglobus* in the LGSF group.

Subsequently, one-way ANOVA was performed to determine the significantly altered microbes in groups. The key feature of microbial alterations induced by LGS, LGSP or LGSF could be summarized as follows: (1) recovery of ALD-mediated abnormal changes; (2) specifically enrichment of certain gut microbes. In the ALD group, the proportion of *Coriobacteriaceae-UCG-002* (Fig. 6B) was decreased significantly, while that of *Parasutterella* (Fig. 6C), *Romboutsia* (Fig. 6D), and *Escherichia-shigella* (Fig. 6E) was significantly increased. Notably, the treatment of LGS, LGSP or LGSF could at least partially recover these ALD-mediated changes. LGSF significantly increased the *Coriobacteriaceae-UCG-002* abundance in ALD mice (Fig. 6B). LGS, LGSP and LGSF all significantly decreased ALD-induced increase of *Parasutterella* and *Romboutsia* (Fig. 6C, D). LGS and LGSF could reverse the ALD-mediated enrichment of *Escherichia-shigella* (Fig. 6E). Furthermore, certain gut microbes were remarkably enriched after treatment of LGS, LGSP or LGSF: *Akkermansia* (Fig. 6F) in MTDX group, *Faecalibaculum* (Fig. 6G) and *Lactobacillus* (Fig. 6H) in the LGS group, *Bacteroides* (Fig. 6I), and *Enterorhabdus* (Fig. 6J) in the LGSP group, and *Monoglobus* (Fig. 6K) in the LGSF group. With a closer look, the *Bacillus* (Fig. 6L) were also significantly increased in LGS and LGSP groups, and *Bifidobacterium* (Fig. 6M) was enriched in the LGS group. The discrepancy in the effect of LGS, LGSP and LGSF may be due to their specific regulation of gut microbes or microbial interaction.

These results suggest that the microbial changes induced by ALD can be restored by LGS, LGSP, and LGSF treatment, and some of them are specific microbes in response to them.

### LGS, LGSP, and LGSF promoted the proliferation of specific bacterial strains in vitro

Based on the microbial analysis, there were specifically altered gut microbes in the LGS, LGSP, and LGSF groups, including the *Faecalibaculum*, *Lactobacillus*, *Bacteroides*, *Enterorhabdus*, *Monoglobus*, *Bacillus*, and *Bifidobacterium*. To see whether there were specific bacterial strains, we investigated the species level alterations. As can be seen from Fig. S9A, several uncultured or unclassified strains were identified in these genera. In particular, the *Bacteroides* spp., *B. sartorii*, was found significantly elevated in the LGSP group (Fig. S9B), and the *Dubosiella* spp., *D. newyorkensis*, was significantly increased in the LGS and LGSP groups (Fig. S9C).

To further investigate the direct effect of LGS and its fractions on specific bacteria strains, in vitro culture was conducted. In addition to the *B. sartorii* and *D. newyorkensis*, the selected bacteria also included the *Lactobacillus*, *Bifidobacterium* strains (*B. adolescentis*, and *B. bifidum*), and *Bacillus* strain (*B. coagulans*), as there have been reports showing the anti-ALD effect of these strains [40, 41]. In order to avoid the interference of glucose in the experimental results, in this experiment, sugar-free culture medium was used in the incubation, ensuring that main carbon source was from the supplemented LGS, LGSP, or LGSF.

**Fig. 7:**
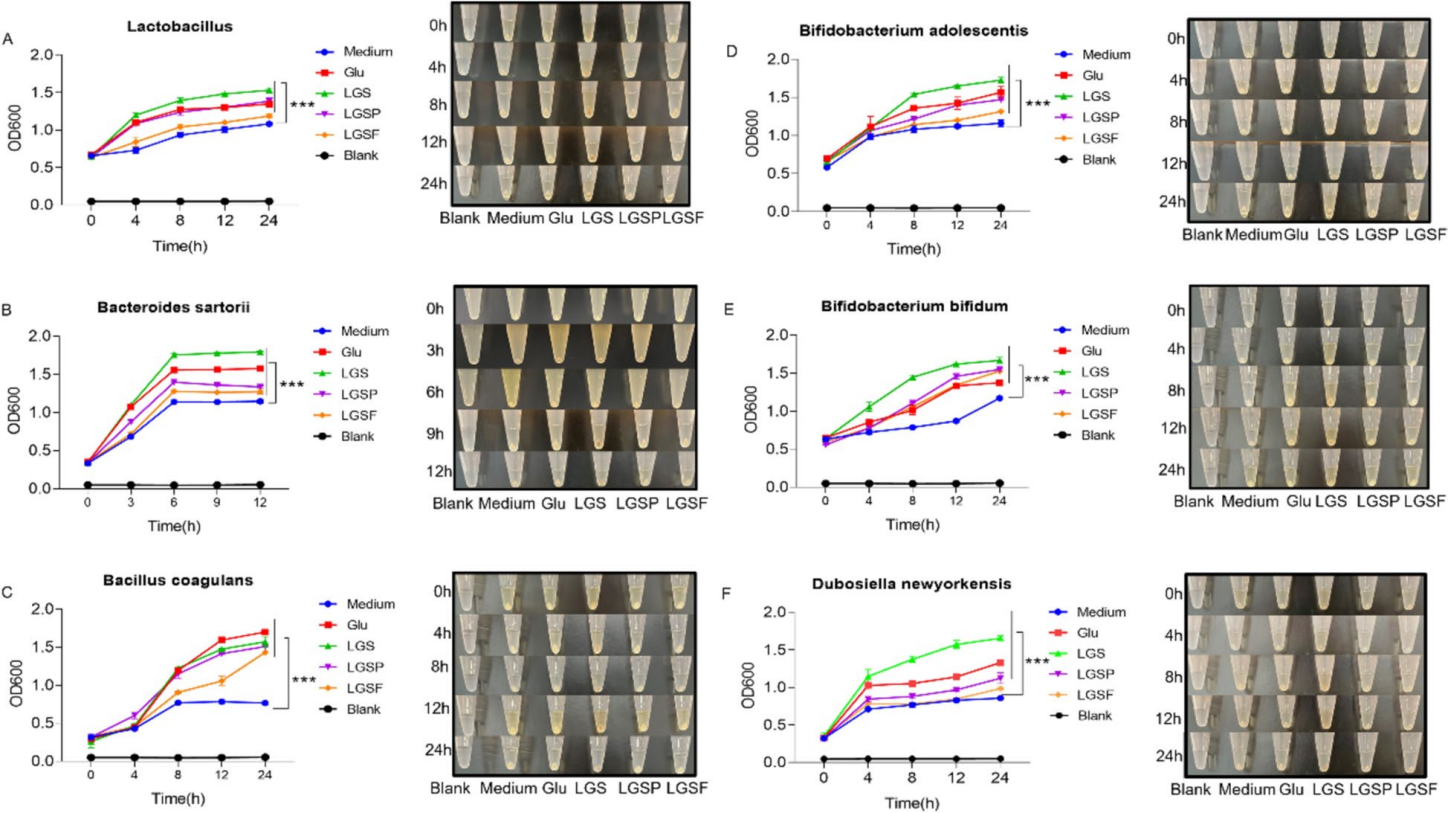
LGS and its active fractions (LGSP and LGSF) promoted the in vitro growth of specific bacterial strains. **A** Growth curve of *Lactobacillus*. **B** Growth curve of *Bacteroides sartorii*. **C** Growth curve of *Bacillus coagulans*. **D** Growth curve of *Bifidobacterium adolescentis*. **E** Growth curve of *Bifidobacterium bifidum*. **F** Growth curve of *Dubosiella newyorkensis*. Glucose (Glu) was used as positive control throughout the experiments. *** *P* < 0.001 vs. medium group; one-way ANOVA with a post hoc Tukey test

The results showed that LGS and LGSP, but not the LGSF, significantly promoted the growth of *Lactobacillus* (Fig. 7A). Similarly, LGS and LGSP significantly increased the proliferation of *B. sartorii*, and LGSF had a minimal effect (Fig. 7B). LGS, LGSP and LGSF all promoted the growth of *B. coagulans* (Fig. 7C), *B. adolescentis* (Fig. 7D), and *B. bifidum* (Fig. 7E). LGS and LGSP promoted the growth of *D. newyorkensis*, but the LGSF had no effect (Fig. 7F). Overall, the promoting effect of LGSF on most of bacteria strains, except the *B. coagulans*, was smaller than that of LGS and LGSP. This may be due to the fact that both LGS and LGSP contained rich carbon source for bacterial growth. Furthermore, the in vitro finding was not exactly consistent with the in vivo results. The probable reason is that flavonoids prevent harmful bacteria from growing and reduce the competitive pressure in the gut [42], thus creating favorable conditions for the growth of probiotics, while in vitro incubation is a single microbe proliferation experiment, which ignores the bacteria-to-bacteria interactions.

Taken together, LGS, LGSP, and LGSF promoted the proliferation of specific bacterial strains in vitro.

## Discussion

The liver is integral to numerous physiological processes, mainly responsible for the synthesis and metabolism of substances, biotransformation, immunity and tolerance to maintain the balance of the microenvironment [43]. The development of ALD encompasses hepatic steatosis, and oxidative stress. Additionally, it involves the direct hepatotoxic effects of ethanol metabolism, inflammation triggered by cytokines and chemokines, intestinal dysfunction as well as gut dysbiosis [44]. In this study, we, for the first time, investigated the underlying mechanisms for the anti-ALD effect of LGS through the view of gut-liver axis, and explored the potential active fractions of LGSP and LGSF.

Previously, we thoroughly investigated the chemical constituents of LGS. It was found that flavonoids and polysaccharides were predominant constituents [18, 24]. Firstly, there was a high proportion of polysaccharides in LGS, representing 17.94 ± 0.28 mg/mL. Notably, the in vitro anti-inflammatory, antioxidant and prebiotic abilities have been demonstrated [24]. Furthermore, in our previous report, 162 small molecule compounds were identified from LGS using ultra-performance liquid chromatography tandem quadrupole time-of-flight mass spectrometry, with a total of 91 flavonoids characterized [18]. The identified flavonoids included puerarin, quercetin, daidzein, dihydromyricetin, rutin and so on. Some of these flavonoids derived from LGS have been demonstrated with positive anti-ALD effects [18, 26, 38, 45]. In this study, the total flavonoid content in LGS was determined as 46 mg/mL, representing a large proportion. Therefore, we speculate that flavonoids and polysaccharides are potentially important chemical basis for the anti-ALD effect of LGS. Moreover, it has been acknowledged that Chinese medicines have the features of multiple constituents and multiple targets. The material basis of Chinese medicines is always not single compound but mostly the structurally similar fractions. In this regard, in this study, we explored the protective effect of LGS and its active fractions of LGSP and LGSF.

We established the ALD model in mice based on previously reported NIAAA method [25]. This model is characterized by significant and stable liver damage. Consistent with previous reports [46], our mouse model of ALD well simulated liver damage (fat accumulation in hepatocytes, destruction of hepatocyte structure) caused by alcohol intake, and various biochemical indicators (TG, TC, LDL, HDL, AST, ALT, IL-1β, IL-6, TNF-α, ADH and ALDH) also changed accordingly. Notably, it was demonstrated that LGS exerted anti-ALD effect in mice through multiple actions including alleviation of hyperlipidemia (reduced TC, TG and LDL; increased HDL) and lipid accumulation in liver (Oil red O stain), reduction of inflammation (reduced IL-1β, IL-6 and TNF-α), and enhancement of ethanol metabolism (increased activity of ADH and ALDH). LGSP and LGSF also showed a protective effect, however, they had some different impacts on some of indices. For example, LGSP did not lower the alcohol-induced AST elevation. LGSF did not reduced the expression of IL-1β, and TNF-α, and did not enhance the activity of ADH and ALDH, which was contrary to the effect of LGSP.

An increasing body of research suggests a robust relationship between the gut-liver axis and the progression of ALD. The gut-liver axis describes the reciprocal interaction between the gut, its microbiota, and the liver, the portal vein acts as a bridge between the intestine and the liver, transporting gut-derived products directly to the liver, where it secretes bile and antibodies from the liver into the gut [47]. Studies have shown that excessive alcohol intake can influence the structure of gut microbiota and cause an imbalance in the intestinal ecology, which can lead to intestinal barrier dysfunction [48]. The consequence of increased intestinal permeability is the facilitation of gut bacteria and their metabolites to transit into the liver through the gut-liver axis. It is noteworthy that the release of endotoxins can activate immune cells in the liver, such as Kupffer cells, triggering an inflammatory response and causing liver impairment [49, 50]. Therefore, the development of ALD needs to be blocked in many ways, the first is to regulate lipid metabolism disorders and improve liver fat accumulation, and the second is to restore the balance of the gut microbiota for repairing the gut barrier and reducing endotoxin secretion. In this study, we thus further considered whether LGS, LGSP, and LGSF could restore intestinal barrier function, support the restoration of intestinal epithelial cells, and recover gut dysbiosis.

Animal experiments demonstrated that LGS, LGSP and LGSF have shown positive intestinal epithelial cell protection and the effect of restoring the intestinal barrier. Therefore, we further applied the 3D culture of small intestinal organoids to directly study the effects of LGS, LGSP, and LGSF on intestinal function and structure. Under pathological conditions, LGS, LGSP, and LGSF were found to enhance the repair of injured intestinal organoids, reduce intestinal epithelial cell apoptosis, and promote the proliferation and differentiation of impaired epithelial cells. Supplementation of LGS, LGSP, and LGSF, especially LGSF could potentially serve as a viable treatment for alcohol-induced liver damage via improving gut barrier dysfunction.

Gut microbiota are crucial in maintaining gut health, and increasing evidence suggests a correlation between the severity of ALD and gut dysbiosis, indicating a bidirectional relationship between the two [40]. In our study, after drug intervention, a number of specific gut bacterial species were identified through microbiome analysis, which may contribute to the improvement of ALD. From the PCoA analysis, it was shown that the microbial structure in LGSF group was similar to that in the control group, and that in the LGS and LGSP was different than the ALD group. This indicates that LGS, LGSP and LGSF greatly impact gut microbiota in ALD mice. Further analysis demonstrated that LGS, LGSP and LGSF alleviated gut dysbiosis in ALD mice. This was evidenced by the key feature of microbial alterations induced by LGS, LGSP or LGSF: (1) recovery of ALD-mediated abnormal changes; (2) specifically enrichment of certain gut microbes. We demonstrated that in ALD mice, the abundance of *Coriobacteriaceae-UCG-002* was decreased, while that of *Parasutterella*, *Romboutsia*, and *Escherichia-shigella* were increased. *Parasutterella*, identified as a pathogenic bacterium, is associated with enteritis and sepsis [30], and is found enriched in ALD previously [51]. *Romboutsia* is associated with fat metabolism and is generally considered harmful, easily leading to an imbalance in the gut flora [52, 53]. *Escherichia-Shigella* in Gut is considered as an opportunistic pathogen [41]. Notably, LGS, LGSP or LGSF could at least partially reverse these alcohol-mediated alterations, indicating a favorable effect on gut microbiota balance.

Interestingly, LGS, LGSP or LGSF specifically induced the increase of some bacteria in the gut, including *Faecalibaculum*, *Lactobacillus*, *Bacteroides*, *Enterorhabdus*, *Monoglobus*, *Bacillus*, and *Bifidobacterium*. Moreover, at species level, two known strains *Bacteroides sartorii* and *Dubosiella newyorkensis* were found increased in LGS and/or LGSP groups. Some strains of *Faecalibaculum* have been demonstrated with beneficial effect on host. For example, *Faecalibaculum rodentium* could maintain eosinophil-dependent gut epithelial hemostasis [54]. *Lactobacillus* spp. has the potential to bolster intestinal barrier integrity and ameliorate ALD [46]. As intestinal commensals, *Bacteroides* primarily functions in the metabolism of polysaccharides and oligosaccharides, supplying nutrients and vitamins to the host [55]. It was reported that dietary fiber alleviated ALD in mice via promoting *Bacteroides acidifaciens* [56]. *Enterorhabdus*, as a potentially beneficial bacterial genus, may be used as a new type of microflora to improve lipid metabolism [57]. Li et al. compared the gut microbial structure between healthy control and hepatitis B virus-induced liver disease and found that *Monoglobus* was enriched in healthy control [58]. Moreover, it has been demonstrated that *Bacillus subtilis* attenuated ALD by regulating intestinal integrity and gut microbiota in mice [59]. *Bifidobacterium* strains have long been proved with varied health benefit [60]. Therefore, it is speculated that LGS and its active fractions (LGSP and LGSF) exert a beneficial effect on gut microbiota structure in ALD mice. To further verify whether the treatment can produce specific intestinal microbiota modulation, we selected several bacterial strains for in vitro incubation, including the *Lactobacillus*, *Dubosiella newyorkensis*, *Bifidobacterium adolescentis*, *Bifidobacterium bifidum*, *Bacteroides sartorii* and *Bacillus coagulans*, which have been potentially associated with the improvement of ALD [61, 62]. The results demonstrated that LGS, LGSP, and LGSF promoted the proliferation of specific bacterial strains in vitro.

In summary, LGS had a therapeutic effect on ALD mice, and the LGSP and LGSF were its active constituents. LGS and its active fractions preferentially regulated the gut-liver axis via specifically promoting gut epithelial barrier and modulating gut microbiota structure. The main limitation of current study included the following: (1) LGS had multi-functions in relieving ALD, however, the exact contribution of these targets remains to be elucidated. (2) whether the identified bacteria responded to LGS treatment had a protective effect on ALD is unclear, which requires future investigation.

## Conclusions

**Fig. 8:**
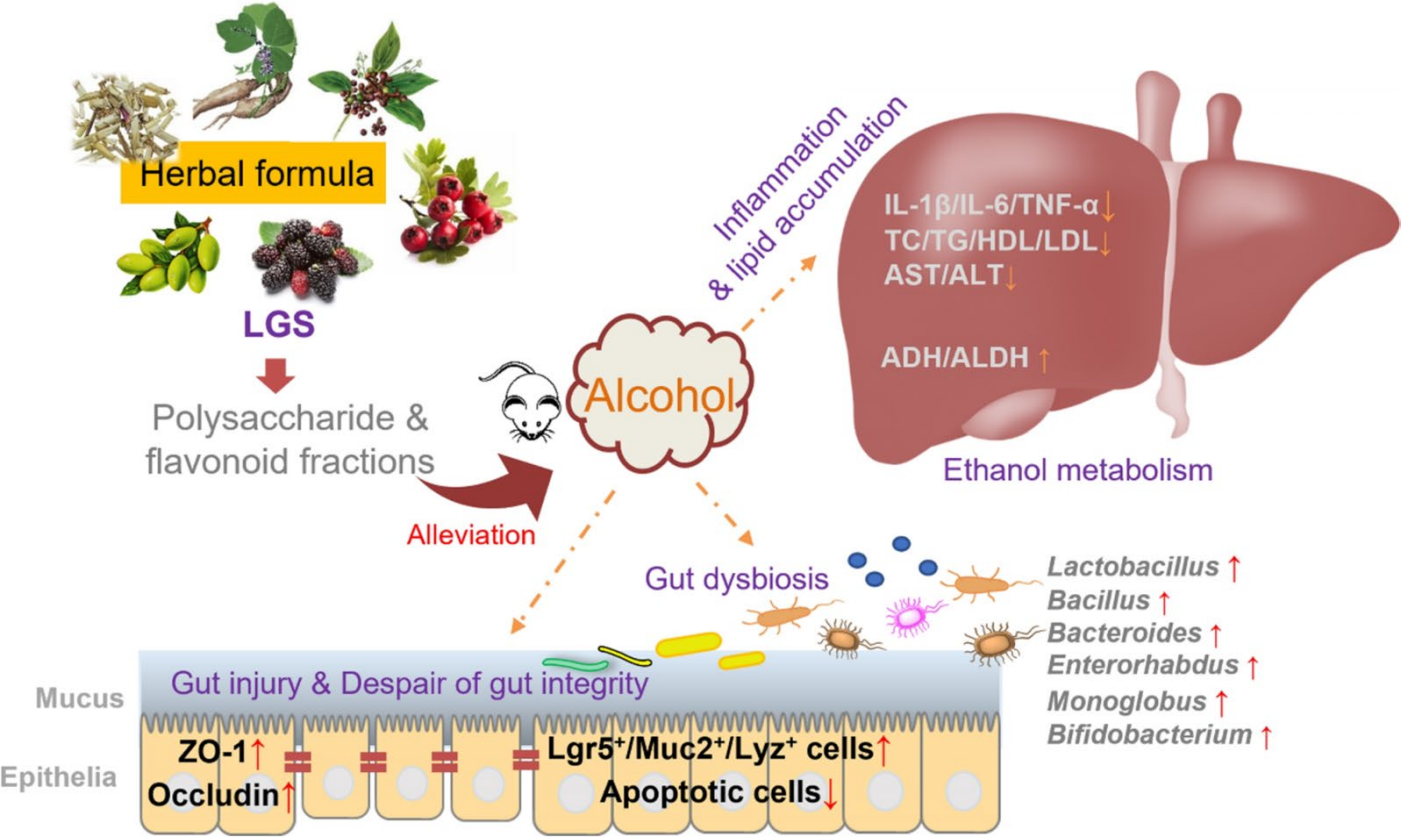
Schematic illustration of the protective effect of LGS and its active fractions (LGSP and LGSF) on ALD through modulating the gut-liver axis

In the present work, the traditional Chinese medicinal formula LGS was demonstrated with significant alleviating effect on ALD in mice through attenuating alcoholic liver injury and inflammation, reducing liver steatosis and enhancing alcohol metabolism. Furthermore, LGS exerted a favorable effect on maintaining gut homeostasis via promoting gut epithelial barrier function and regulating gut microbiota. The polysaccharide and flavonoid fractions, LGSP and LGSF, were identified as the effective constituents responsible for ALD alleviation of LGS. We demonstrated that either LGS or its active fractions (LGSP and LGSF) had a direct protective effect on gut epithelial injury (indicated on intestinal organoid model) and exerted a specific impact on particular bacterial strains (revealed by in vitro incubation). The results of this study successfully elucidate the association between LGS compositions and its anti-ALD efficacy (Fig. 8), which will help with the future development of novel LGS-based anti-ALD drugs.

## Supplementary Information

**Additional file 1.**

## Data Availability

The data supporting this study’s findings are available from the corresponding author upon reasonable request.

## Abbreviations

ALD: Alcoholic liver disease
ALDH: Acetaldehyde dehydrogenase
ALT: Alanine aminotransferase
AST: Aspartate transaminase
BV: Bed volume
CICC: China Center of Industrial Culture Collection
GLP-1: Glucagon-like peptide 1
Glu: Glucose
HDL: High density lipoprotein
H&E: Hematoxylin and eosin
IECs: Intestinal epithelial cells
IF: Immunofluorescence
IHC: Immunohistochemistry
IL-1β: Interleukin-1β
IL-6: Interleukin-6
IOGM: Intesticult™ Organoid Growth Medium
LDL: Low density lipoprotein
LEfSe: Linear discriminant analysis effect size
LGS: Gegen-Sangshen oral liquid
LGSF: The flavonoid fraction of LGS
LGSP: The polysaccharide fraction of LGS
LPS: Lipopolysaccharide
OD: Optical density
PCoA: Principal coordinate analysis
TC: Total cholesterol
TCM: Traditional Chinese Medicine
TG: Triglyceride
TJPs: Tight junction proteins
TNF-α: Tumour necrosis factor-α
ZO-1: Zona occludin 1
