# Supplementary material for: Gegen-Sangshen oral liquid and its active fractions mitigate alcoholic liver disease in mice through repairing intestinal epithelial injury and regulating gut microbiota
Source: Chin Med. 2024 Dec 23;19:175. doi: 10.1186/s13020-024-01049-y (PMC11667864; doi:10.1186/s13020-024-01049-y)
Supplement: Supplementary file 1 — Additional file 1. [file 13020_2024_1049_MOESM1_ESM.docx]

**

**

**Fig. S1. HPLC chromatogram of the flavonoid fraction of LGS. The main peaks were identified with reference standards.**

**
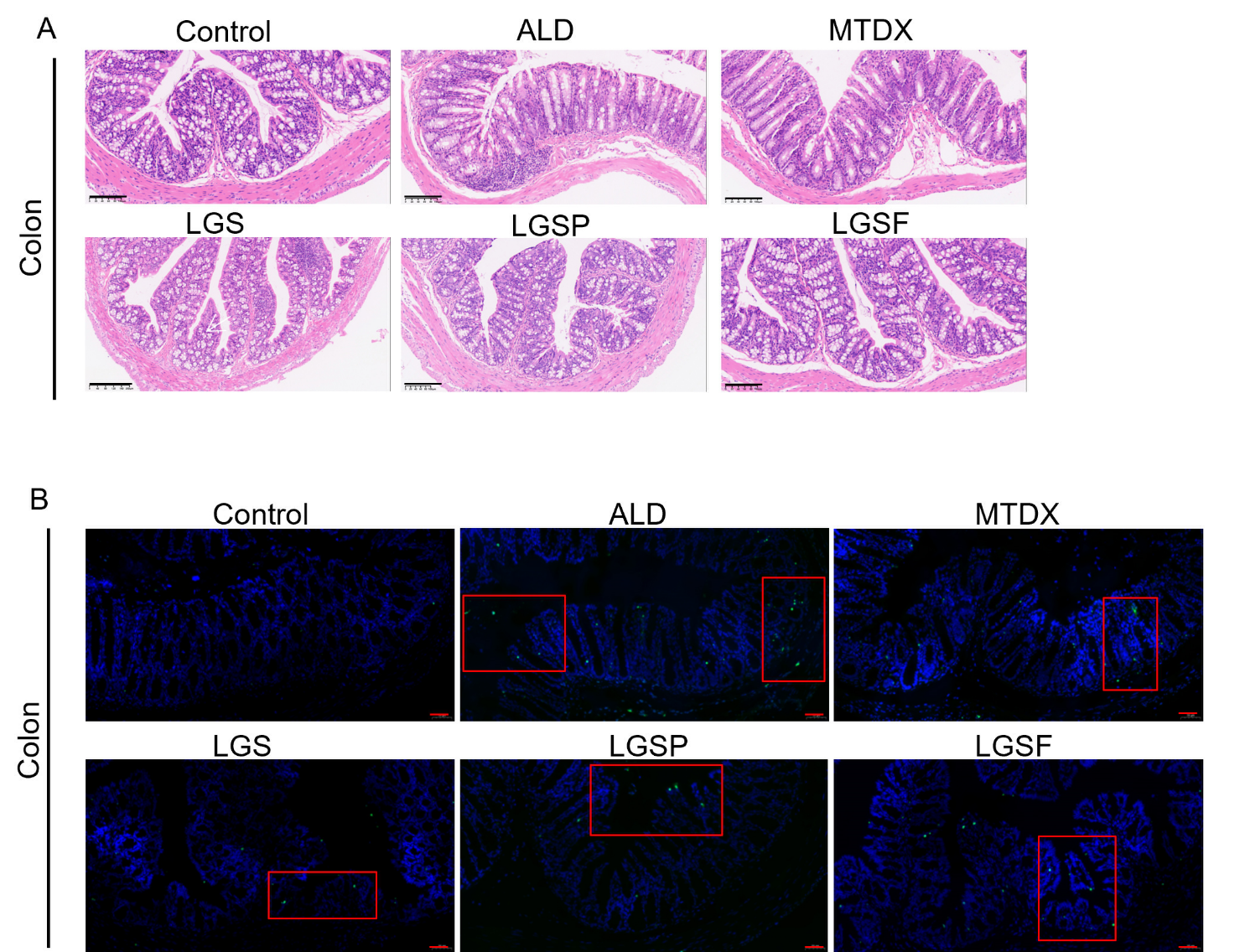
**

**Fig. S2. LGS and its active fractions (LGSP and LGSF) mitigated colonic injury in ALD mice. (A)** H&E staining of colon sections. (**B**) TUNEL-stained colon sections. The inner part of the red box is apoptotic cells.


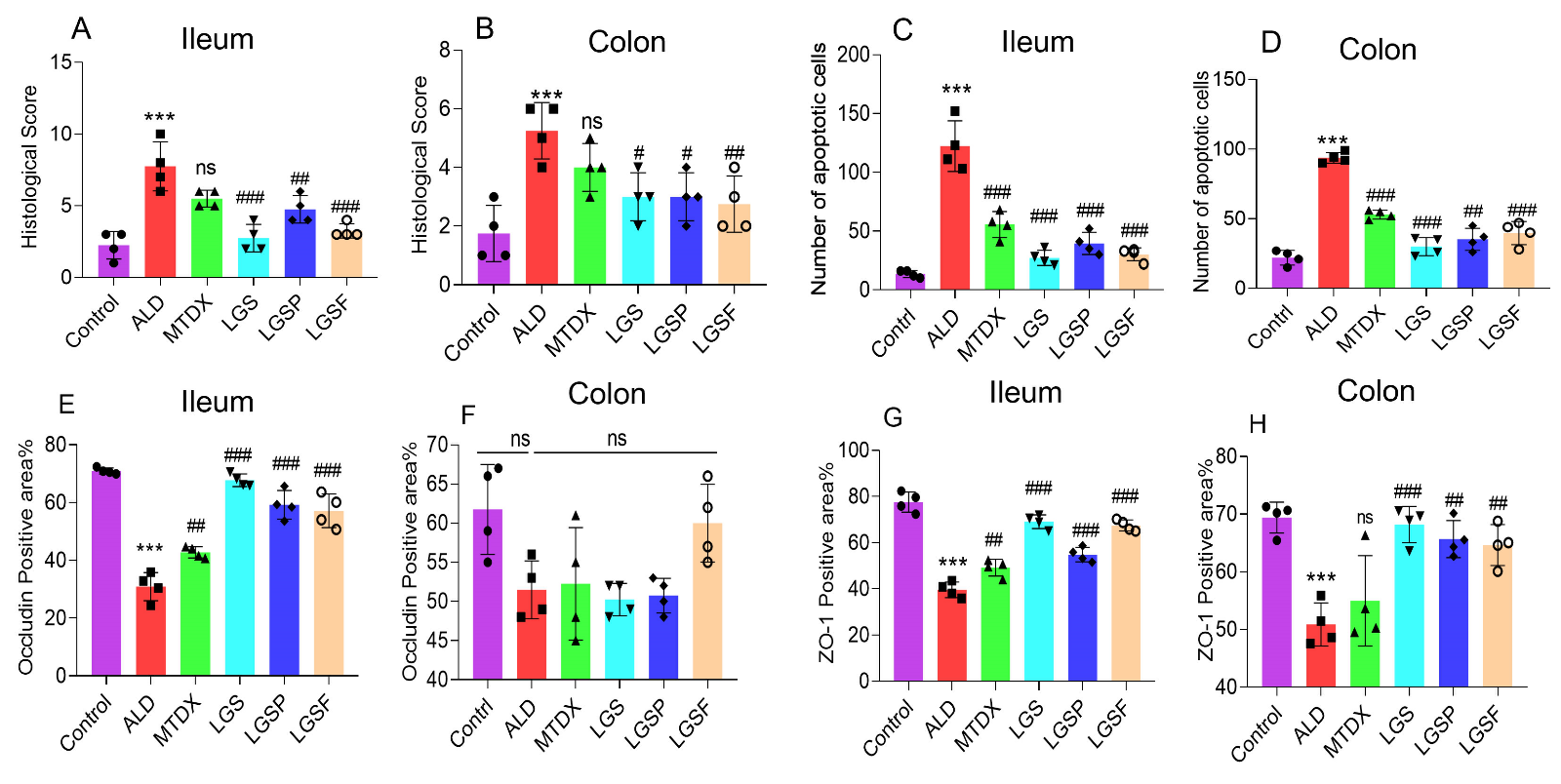


**Fig. S3. (A)** Histopathological score based on H&E sections of ileum. (B) Histopathological score based on H&E sections of colon. (C) Number of TUNEL-positive apoptotic cells in ileum. (D) Number of TUNEL-positive apoptotic cells in colon. (E) Occludin positively stained areas in ileum. (F) Occludin positively stained areas in colon. (G) ZO-1 positively stained area in ileum sections. (H) ZO-1 positively stained area in colon sections. (n=4). ^#^*P* < 0.05, ^##^*P* < 0.01, ^###^*P* < 0.001 *vs* ALD group. ^*^*P* < 0.05, ^**^*P* < 0.01 and ^***^*P* < 0.001 *vs* Control group; ns, non-significant; one-way ANOVA with a post hoc Tukey test.

**Fig. S4. The serum level of LPS in ALD mice.** ^#^*P* < 0.05, ^##^*P* < 0.01, ^###^*P* < 0.001 *vs* ALD group. ^*^*P* < 0.05, ^**^*P* < 0.01 and ^***^*P* < 0.001 *vs* Control group; one-way ANOVA with a post hoc Tukey test.


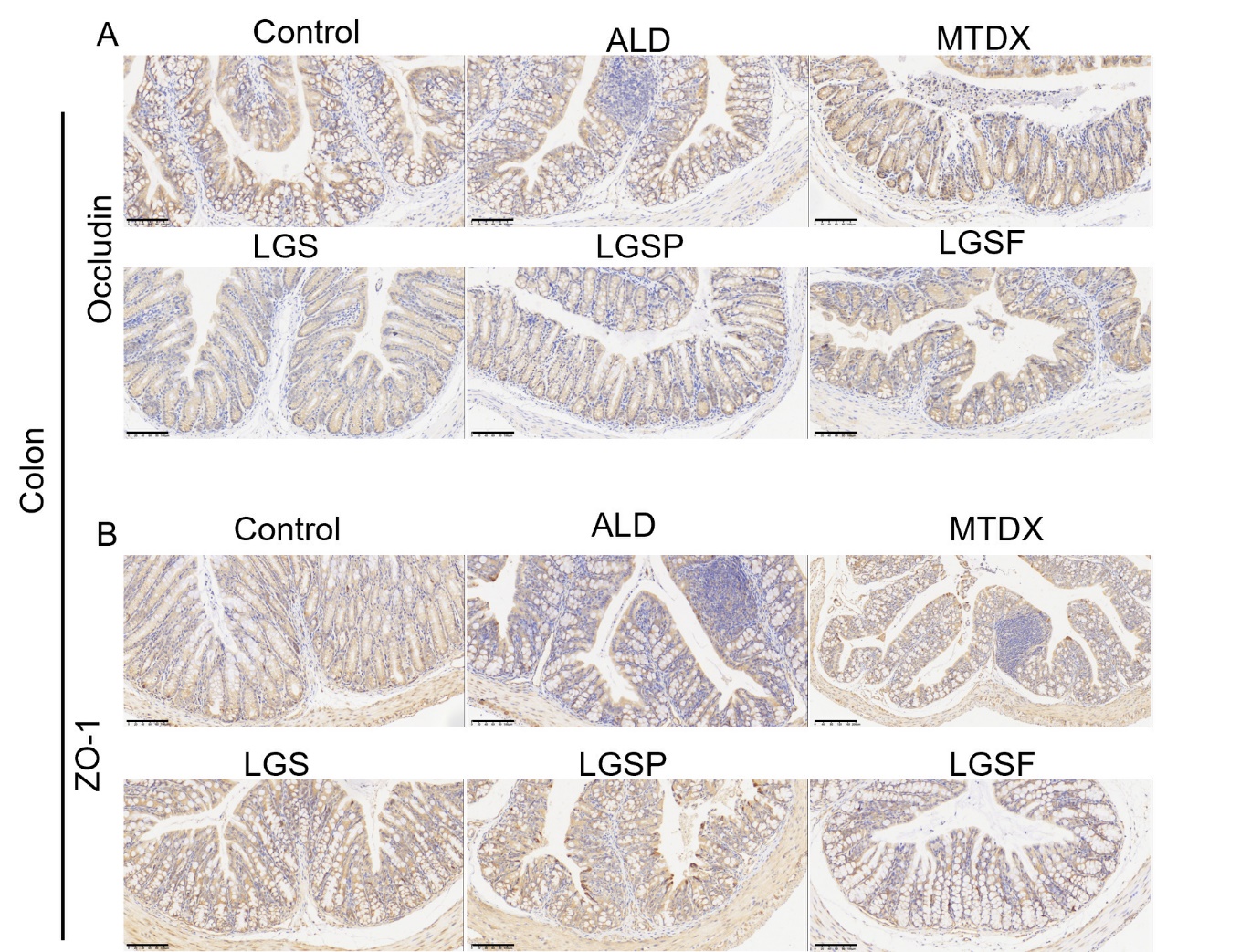


**Fig. S5. Effects of LGS and its active fractions (LGSP and LGSF) on the expression of tight conjunction proteins in colon. (A)** The expression of occludin protein in colon. (**B**) The expression of ZO-1 protein in colon.

**
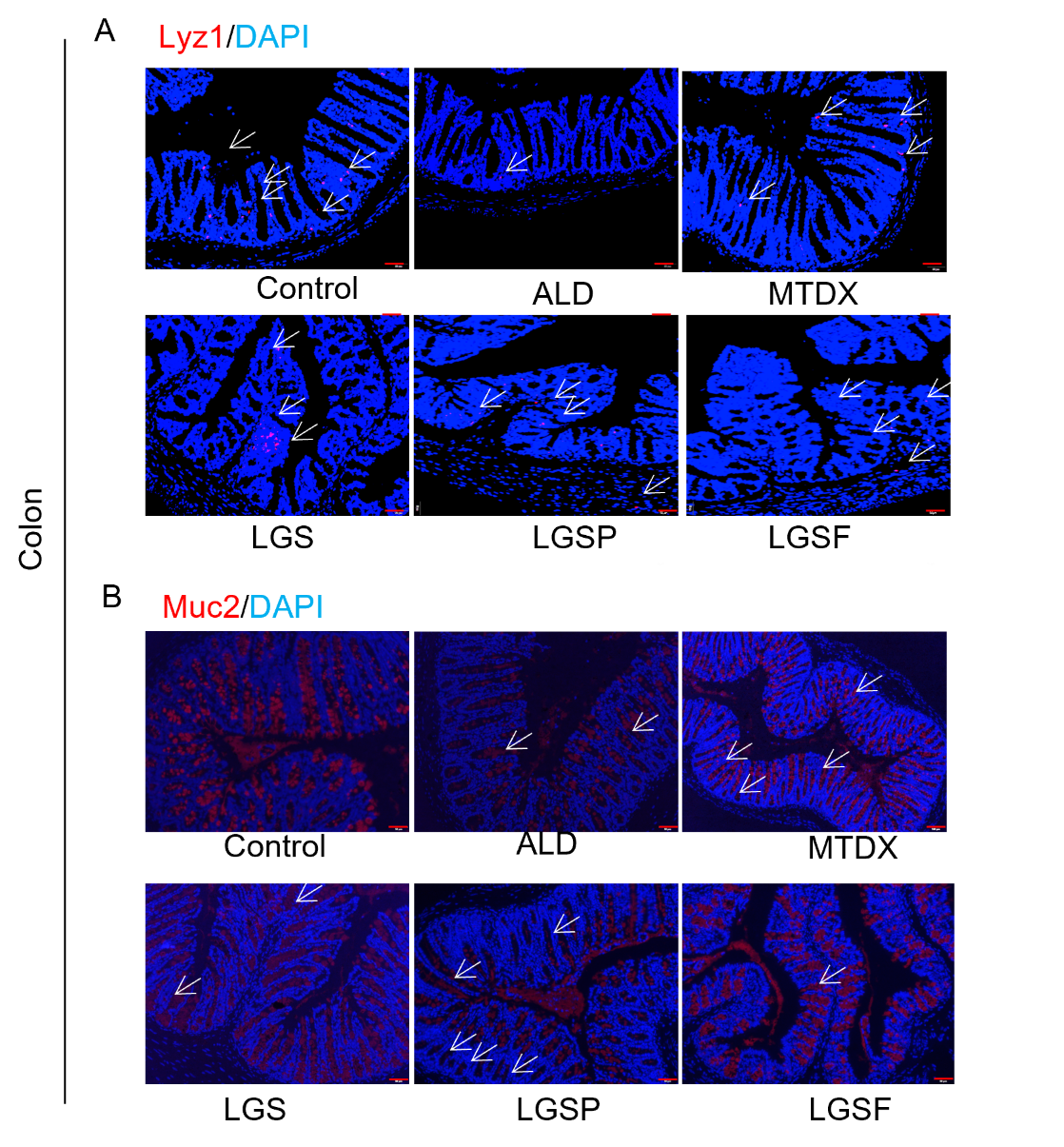
**

**Fig. S6.** **LGS and its active fractions (LGSP and LGSF) promoted intestinal epithelial proliferation in colon of ALD mice. (A)** Immunofluorescence staining of Lyz1^+^ cells in colon. **(B)** Immunofluorescence staining of Muc2^+^ cells in colon.


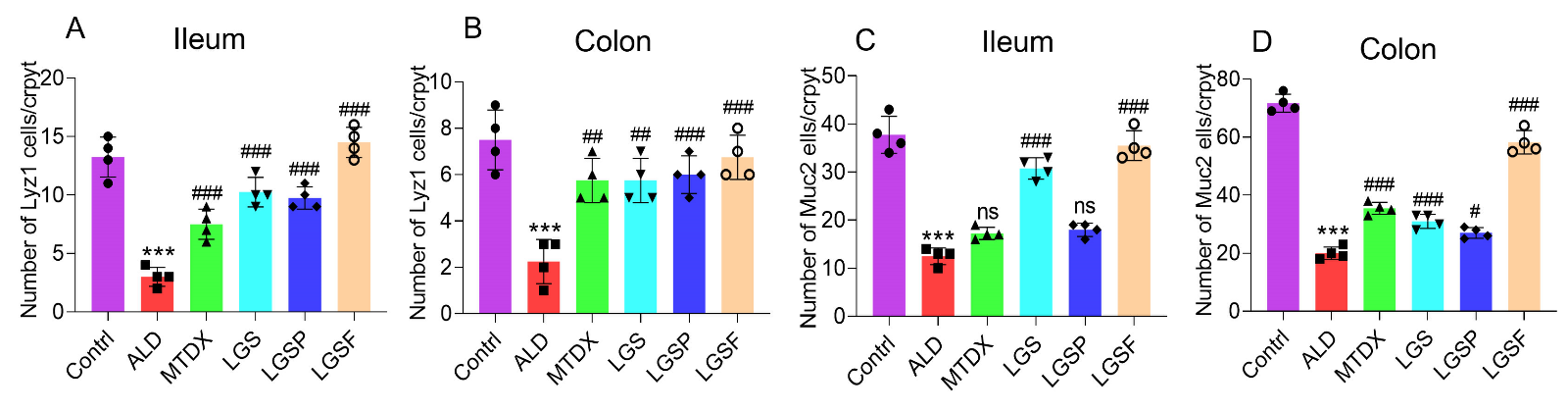


**Fig. S7. (A)** Number of Lyz1-positive cells in ileum. (B) Number of Lyz1-positive cells in colon. (C) Number of Muc2-positive cells in ileum. (D) Number of Muc2-positive cells in colon. N=4. ^#^*P* < 0.05, ^##^*P* < 0.01, ^###^*P* < 0.001 *vs* ALD group. ^*^*P* < 0.05, ^**^*P* < 0.01 and ^***^*P* < 0.001 *vs* Control group; ns, non-significant; one-way ANOVA with a post hoc Tukey test.


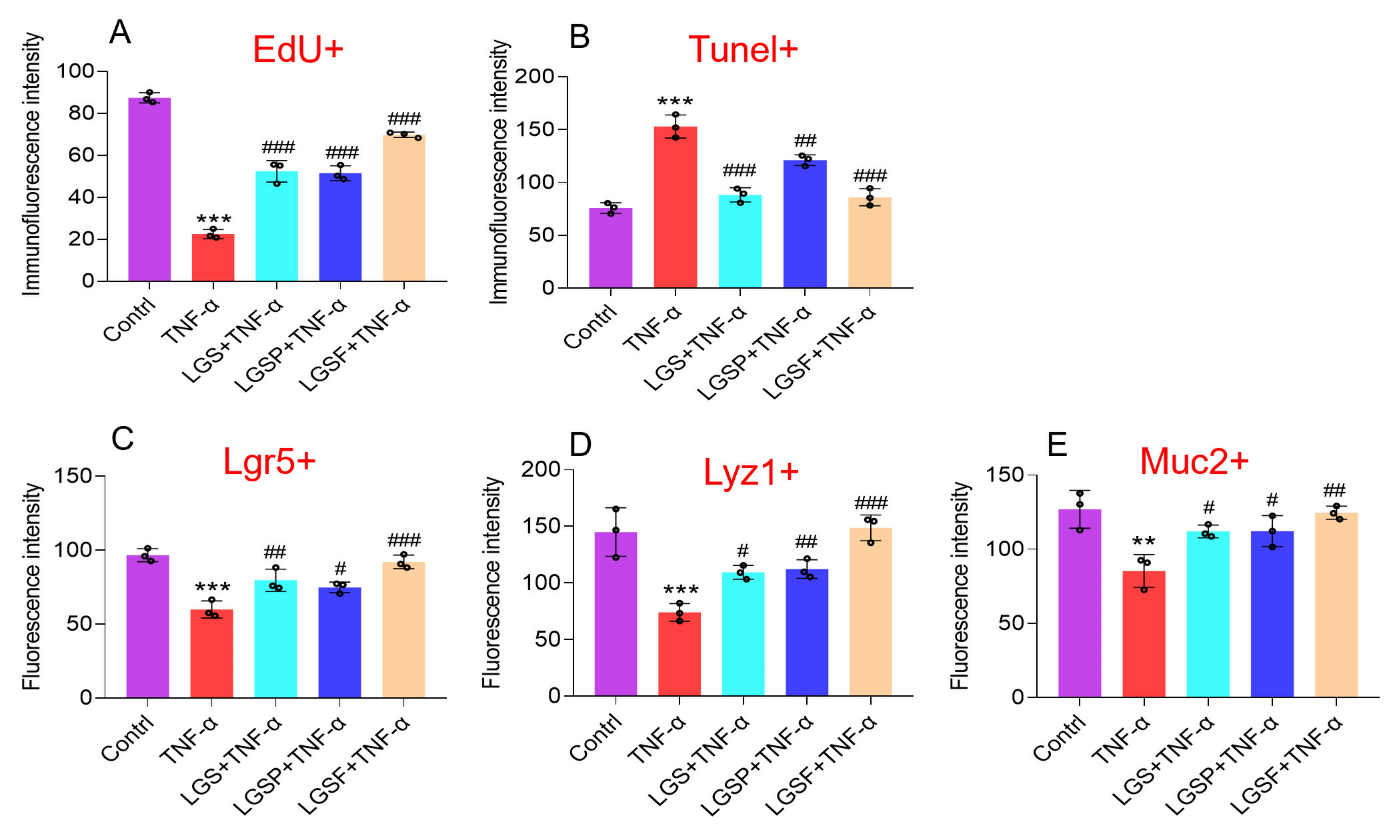


**Fig. S8.** Quantitative analysis of histopathological scores, TUNEL-positive cells, occludin or ZO-1 positively stained area, and number of Lyz1^+^ and Muc2^+^ cells.

**
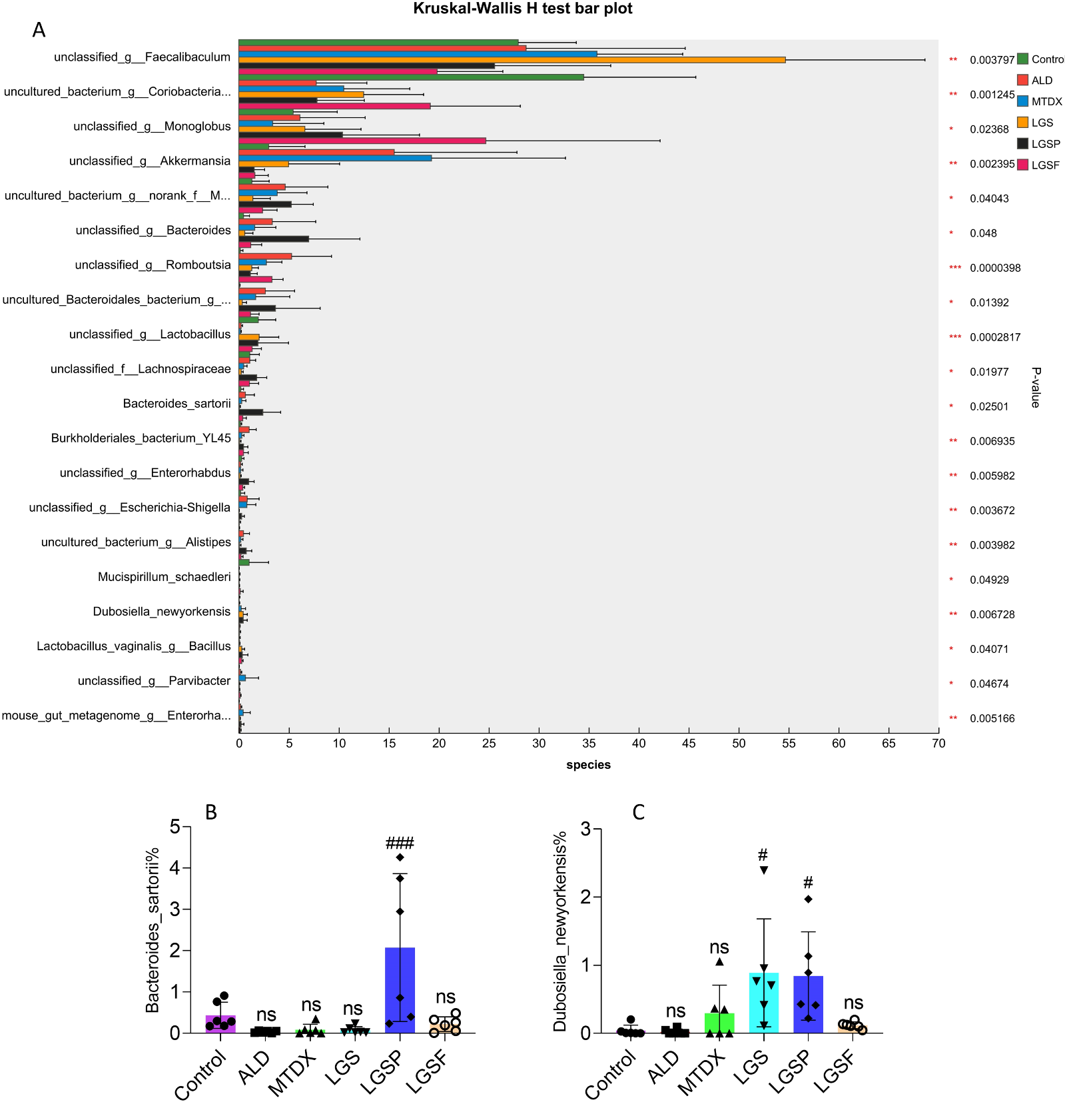
**

**Fig. S9. (A) Kruskai-Wallis H test bar plot of microbial changes at species level.** (B) The relative abundance of *Bacteroides sartorii*. (B) The relative abundance of *Dubosiella newyorkensis*. Ns, non-significant. ^#^*p*<0.05, ^###^*p*<0.001, vs ALD.
